# Characterizing missed identifications and errors in latent fingerprint comparisons using eye-tracking data

**DOI:** 10.1371/journal.pone.0251674

**Published:** 2021-05-24

**Authors:** Thomas A. Busey, Nicholas Heise, R. Austin Hicklin, Bradford T. Ulery, JoAnn Buscaglia

**Affiliations:** 1 Psychological and Brain Sciences, Indiana University, Bloomington, Indiana, United States of America; 2 Intelligence and Analytics, Noblis, Reston, Virginia, United States of America; 3 Research and Support Unit, Federal Bureau of Investigation Laboratory, Quantico, Virginia, United States of America; University of Canberra, AUSTRALIA

## Abstract

Latent fingerprint examiners sometimes come to different conclusions when comparing fingerprints, and eye-gaze behavior may help explain these outcomes. missed identifications (missed IDs) are inconclusive, exclusion, or No Value determinations reached when the consensus of other examiners is an identification. To determine the relation between examiner behavior and missed IDs, we collected eye-gaze data from 121 latent print examiners as they completed a total 1444 difficult (latent-exemplar) comparisons. We extracted metrics from the gaze data that serve as proxies for underlying perceptual and cognitive capacities. We used these metrics to characterize potential mechanisms of missed IDs: Cursory Comparison and Mislocalization. We find that missed IDs are associated with shorter comparison times, fewer regions visited, and fewer attempted correspondences between the compared images. Latent print comparisons resulting in erroneous exclusions (a subset of missed IDs) are also more likely to have fixations in different regions and less accurate correspondence attempts than those comparisons resulting in identifications. We also use our derived metrics to describe one atypical examiner who made six erroneous identifications, four of which were on comparisons intended to be straightforward exclusions. The present work helps identify the degree to which missed IDs can be explained using eye-gaze behavior, and the extent to which missed IDs depend on cognitive and decision-making factors outside the domain of eye-tracking methodologies.

## 1 Introduction

Fingerprint comparisons can be a demanding perceptual and cognitive task, especially in cases where the fingerprints are of poor quality, limited quantity, or both [1–7]. A latent print examiner will collect or receive a latent (friction ridge impression from the fingers, palms, or feet of an unknown subject), determine whether it is of value for comparison, and, if so, compare it against exemplars (prints deliberately collected from known subjects), either from the result of a database search or from a suspect. The examination process relies on features that are selected by the examiner, and the examiner must establish correspondences between the two sets of features in order to make an identification decision. These features typically consist of minutiae, which never correspond precisely due to differences in the deposition process, the recording medium, distortion, and factors such as humidity and surface composition.

Examiners rely on a framework known as ACE-V [8, 9], in which they conduct a side-by-side comparison of the two impressions. They first conduct an analysis of the latent to identify target features or groups (usually a minutia or collection of minutiae) and to determine whether it is of value for comparison. During a subsequent comparison phase, they encode these features into visual working memory. The limits of visual working memory usually only allow a few features in a small spatial region to be encoded. Once these features are encoded, the examiner then makes a rapid eye movement (saccade) to the exemplar impression and searches for a possible corresponding area. Performance in this task may depend on the efficiency and accuracy of this encoding/search/comparison sequence. Although there is no fixed standard in the United States, some examiners use an informal or implicit threshold of 7–12 corresponding minutiae to effect an identification conclusion [4], and thus this encoding/search/comparison sequence must be executed multiple times. This dependence on eye movements between potentially corresponding regions makes eye tracking a suitable technique to measure the underlying perceptual and cognitive processes that support fingerprint comparisons. Recent work by Malhotra et al. [10] tested latent print examiners and used eye gaze to address the relation between eye gaze, minutiae, image clarity, region of interest, and measures of alignment using an Earth Mover Metric, demonstrating the utility of eye-gaze data to address latent print comparison behavior.

After comparison, examiners make one of three evaluation determinations: identification, inconclusive, or exclusion. Historically, examiners did not differentiate between inconclusive and exclusion and reported a not identified conclusion. The term “missed Identification” (missed ID) is used to refer to a failure to make an identification (i.e., No Value, inconclusive, or exclusion) on a comparison identified by other examiners. Missed IDs can have serious implications in casework in that some potential identifications are not being made, and this can be seen as a failure of the forensic examination to deliver a reliable result. It should be noted that missed IDs can also reflect an examiner’s own assessment of their abilities: an inexperienced examiner may be appropriately cautious, but therefore make more inconclusive decisions than the norm. In [1], 4.7% of responses on mated pairs (image pairs where the ground truth is known that they were generated by the same finger) were missed IDs—there defined as exclusion, inconclusive, or No Value determinations on mated image pairs that the majority of examiners identified.

What mechanisms are responsible for missed IDs? Previously, Ulery et al. [7] reviewed erroneous exclusions and reported that they could generally be attributed to four causes. Here we adapt that list to propose four conceptual explanations for non-consensus inconclusive decisions as well as erroneous exclusions:

1. *Cursory Comparison*: Misinterpretation of pattern class (e.g., whorl or loop) or overall ridge flow (the dominant direction of ridges in a region of the impression) resulting in an erroneous exclusion (due to incorrectly determining the pattern class or ridge flow are incompatible), or inconclusive (due to incorrectly assessing that no potentially corresponding areas are present). Missed IDs caused by cursory comparisons would generally be expected to have little or no detailed comparison (i.e., at the minutia level), and be relatively brief.
2. *Mislocalization*: Incorrect anchoring (basing the comparison on an erroneous assumption of which minutiae or regions correspond) or incorrect rotation resulting in an erroneous exclusion or inconclusive decision. In some instances, searched-for regions may be mislocalized (or partially mislocalized, based on some correct and some incorrect correspondences due to incorrect ridge counting or misinterpretation of distortion). Missed IDs caused by mislocalization would be expected to be associated with inaccurate attempts at correspondences.
3. *Invalid Discrepancies*: Correct anchoring and assessments of correspondences that nevertheless result in erroneous exclusions due to incorrectly treating a difference in the impression as a discrepancy (i.e., an actual difference in the skin itself). The eye-gaze data for erroneous exclusions caused by invalid discrepancies would be expected to be very similar to that of trials resulting in correct identifications.
4. *Sufficiency*: Correct anchoring and assessments of features and correspondences that result in inconclusive conclusions due to the examiner’s individual criteria for making identifications, determining that the extent of corresponding information is not sufficient to meet that examiner’s personal threshold (i.e., due to differences in caution or risk tolerance). In an extreme case of missed ID due to lack of sufficiency, an examiner might determine that a latent print is No Value (insufficient features to compare), whereas other examiners declare Of Value, compare, and make identification conclusions. As with the *Invalid Discrepancies* explanation, the eye-gaze data for non-consensus inconclusive conclusions due to sufficiency disagreements would be expected to be very similar to the data from identification conclusions. Note, however, that the two explanations are conceptually different, and one applies to erroneous exclusion conclusions, while the other applies to inconclusive conclusions.

To describe the behavior that is associated with missed IDs, we conducted a large-scale data collection project that involved eye tracking for ~2.5 hours from each of 121 latent print examiners as they conducted a series of fingerprint comparison tasks. Previous work on sufficiency [4] and exclusions [7] assessed examiners’ decisions based on manual markup of corresponding or discrepant features. Such manual markup can only explain a subset of missed IDs: if examiners erroneously excluded (or were inconclusive on) a mated image pair, they often marked nothing at all and therefore provided no indication of where they went wrong. Here, even if examiners fail to mark features or correspondences on missed ID trials, we will still record where their eyes point. This forms the genesis of the current study.

We present analyses of the eye-gaze data at different timescales and levels of abstraction. Our goal with each analysis is to define a *proxy* for a cognitive capacity that may contribute to outcomes, including missed IDs. We use “outcome” to refer to the combination of examiner determination and whether the image pair is mated (e.g., an identification determination on a mated pair results in a true positive (TP) outcome). A proxy for a cognitive capacity can be something as simple as the time taken to complete the task, or as complex as relative alignment of locations in the images that the examiner looked at during the trial. We will derive a *metric* for each proxy, which computes one number per trial, and then associate the values of each metric with various outcomes.

There are several aspects of fingerprint comparisons that suggest that eye tracking may be useful. First, the relevant features (minutiae) are small and typically require foveal viewing. Global ridge flow as encoded using peripheral regions is almost certainly used to guide fixations [11, 12] but is not suitable for the fine details in minutiae. Second, visual targets such as minutiae and ridge elements are not easily transcoded to linguistic or symbolic representations and are therefore subject to the fairly limited capacity and relatively rapid decay exhibited by visual working memory. This means that the examiner will have to periodically refresh the contents of working memory by fixations on the regions he/she determines to be relevant, and we can measure this behavior with an eye tracker. However, first we need to connect the above conceptual explanations for missed IDs to eye-gaze data.

A missed ID can result from both large-scale and small-scale examination disagreements, some of which are differentiable based on eye-gaze data. It is useful to discuss the strengths and limitations of eye-tracking data for our purposes, to demonstrate what is achievable with eye-tracking analyses and to connect with prior literature on missed IDs. Examiners who reach different conclusions based on large-scale examination differences should generally be differentiable based on eye-gaze behavior, and we will explore the evidence that is consistent with these explanations. Eye-gaze behavior may or may not have the resolution to be able to assess small-scale examination differences.

Large-scale examination differences involve disagreements regarding pattern class, ridge flow, and regions of interest within the images [13]. Large-scale examination disagreements that may explain different examiners’ conclusions can be summarized as follows:

- Do examiners interpret the pattern and ridge flow the same way?
- Do examiners use the same areas of the impressions (i.e., regions of interest)?
- Do examiners find regions of correspondence?

Each of these represent proposed cognitive mechanisms. Although interpretation is not generally measurable using eye gaze, we can readily estimate regions of interest and correspondence from the eye-gaze data using several inferential techniques.

Small-scale examination differences are disagreements regarding specific ridges and ridge features, and in some cases may explain differences in examiners’ conclusions:

- Within a given area, do they see the same minutiae and other ridge features, and do examiners interpret them in the same way? (e.g., Do examiners agree on what should be considered artifacts of the images vs. features of the friction ridge skin?)
- Do examiners agree on which specific features correspond?

The extent to which examination differences are detectable using eye-gaze data is primarily limited by the resolution of eye tracking. Eye-gaze techniques generally have a spatial error that is larger than the scale of minutiae, making it difficult to determine the specific features examiners might rely on, thereby limiting the ability to measure or make inferences about small-scale examination differences. Large-scale examination differences, however, should generally be differentiable based on eye-gaze behavior: although interpretation is not generally measurable using eye gaze, we can readily estimate regions of interest and correspondence from the eye-gaze data using several inferential techniques.

Missed IDs that are attributable to large-scale examination differences may at least in concept be assessed by the eye gaze in these ways:

Do they interpret the pattern and ridge flow the same way?
  - Those missed IDs (both exclusions and inconclusives) based predominantly on misinterpretations of the pattern class and/or overall ridge flow may be holistic decisions in which no areas of the impressions are considered as potential correspondences. These trials may have relatively little comparison-like behavior and relatively short comparison times. See image pair X in Fig 3 of [1] for an extreme example. The shortest comparison times are disproportionately missed IDs.
Do they use the same areas of the impressions (i.e., regions of interest)?
  - For latent impressions that are highly distorted, smeared, or may have been the result of multiple superimposed impressions, the region of interest is not always apparent, and requires expertise—and therefore can be expected to vary among examiners. Using different areas of images in comparison would not necessarily result in differences in conclusions: for example, one examiner may limit comparison to a small, relatively high-clarity area whereas another may extend into low-clarity areas, but both reach the same conclusion.
Do they find regions of correspondence?
  - The number of regions that examiners assess to be in correspondence (or the total area of correspondence) is likely to be the primary basis for an identification decision, and therefore we would expect fewer regions of correspondence to be associated with missed IDs.

### 1.1 Research questions

To address the above possible explanations for missed IDs, we organize our research questions into the following categories:

1. To what degree do our metrics support a *Cursory Comparison* explanation for missed IDs? We will look at comparison time as well as a metric that describes the subphases of eye-gaze behavior in terms of scanning vs. detail-oriented movements.
2. How is eye-gaze behavior related to making correspondences between two impressions associated with outcomes? Fundamentally, a latent print comparison involves locating regions of possible correspondence in the two impressions, and we develop a metric that measures the spatial accuracy of such correspondence attempts to assess the evidence for a *Mislocalization* explanation for missed IDs.
3. How is image region selection associated with different outcomes? This analysis addresses metrics such as the proportion of the image fixated by examiners, the spatial spread of the fixations on the latent impression, image clarity, and an Earth Mover metric that computes the distance between fixation sets to address the degree to which examiners look in similar locations. This relates to the ‘region of interest’ large-scale examination difference.
4. Does eye-gaze data provide an explanation for erroneous identifications? Although our focus is primarily on missed IDs, we demonstrate the potential utility of eye-gaze data to explain erroneous identifications. As is demonstrated by the data, this relates to the ‘regions of correspondence’ large-scale examination difference, as these errors appear to result from erroneous correspondences by the participant.

As we associate our metrics with outcomes, we will discuss the support for *Cursory Comparison*, *Mislocalization*, *Invalid Discrepancies*, and *Sufficiency* explanations for missed IDs. Note that these mechanisms may not be disjoint, in the sense that an early mislocalization could lead to a cursory comparison, or the basis for the mislocalization might be an incorrect perceptual interpretation (*Invalid Discrepancy*).

## 2 Materials and methods

### 2.1 Ethics statement

This study was reviewed and approved by the Institutional Review Board of the US Federal Bureau of Investigation (FBI) under docket number 354–16. In addition to the use of a data-coding system to ensure participant anonymity, written informed consent was obtained from all participants.

We collected eye-gaze data from latent print examiners while performing fingerprint comparisons and related tasks. The primary focus of the experimental design was to evaluate examiner eye-gaze behavior when conducting difficult comparisons of latent fingerprints with exemplar fingerprints (“latent-exemplar” comparisons). These were interspersed with exemplar-exemplar comparisons that served to provide baseline data on how examiners conduct easy comparisons (as well as a respite from the difficult latent-exemplar comparisons).

### 2.2 Fingerprint data

The dataset included 45 latent-exemplar image pairs (25 mated and 20 nonmated). These image pairs were selected from the image pairs previously used in the “black box” [1] and “white box” [4] studies, based on the responses received in those studies. Thirty-eight of the image pairs were selected based on low reproducibility of conclusions and/or erroneous conclusions in those previous studies. Seven image pairs were selected based on unanimous conclusions in those previous studies, with the intent of providing an archetypal baseline for consensus conclusions; however, as reported in [14], after assigning each of these to an additional 25–34 examiners, only one remained unanimous (see S1 Appendix for more detail). Because the image pairs were specifically selected to assess reproducibility of examiner conclusions, the dataset was explicitly not intended to be representative of casework in general. More mates than nonmates were selected to focus on understanding missed IDs.

The dataset also included 18 exemplar-exemplar image pairs (8 mated and 10 nonmated), selected to assess how very easy comparisons are conducted. All of the exemplars were very high quality, from the “ULW Ground Truth” dataset (1000ppi scans of the same images as used in the NIST Special Database 27). The mates were expected to be obvious IDs. The nonmates were expected to be obvious exclusions: six of the nonmates were unrelated pattern classes (e.g., whorl vs. loop), and four were superficially similar pattern classes (e.g., left loop against left loop).

### 2.3 Participation

Participation was open to practicing latent print examiners who are currently doing casework or have done casework within the last year. Participants gave informed consent after reviewing a human subject consent form approved by the Federal Bureau of Investigation Institutional Review Board prior to the start of the study. A total of 122 examiners participated; data from one examiner was unusable (due to a corrupt file), resulting in 121 examiners used in analyses. Of the 122 participants, 39% were from federal agencies, 31% state, 22% local, 5% international, and 2% private. 79% were from accredited labs. 76% had five or more years of experience as a latent print examiner; none had less than one year. 19% wore glasses, 29% had contact lenses, and 7% had LASIK. Most participants (64%) had 5–14 years of experience; 12% had 15 or more years, and 24% had 1–4 years. Participants were assured that their results would remain anonymous; a coding system was used to ensure anonymity during our analyses and in reporting. We did not ask about age or gender. Complete survey results are reported in the Supporting Information of Hicklin et al. [12].

### 2.4 Test procedure

Each examiner was assigned 15 latent-exemplar fingerprint comparisons and 6 exemplar-exemplar fingerprint comparisons, interspersed with three types of directed tasks. In addition to the latent-exemplar comparisons that are the focus of this paper, the participants were assigned find-the-target directed tasks, reported in [12], and ridge following and ridge counting tasks, which are not yet reported. Testing occurred in June-August 2016 in six locations in the US. Participants were provided with written instructions prior to the test (summarized in Appendix SI-3.2 in S3 Appendix). An experimenter then verbally summarized the instructions and answered any questions. Participants were requested to continue testing for two hours or until all of the assigned trials were completed; however, participants were permitted to stop early or continue after the two-hour time period.

On each trial, examiners first completed an analysis of the latent print, which was the only image displayed. They were allowed to translate, rescale, and mark relevant features using the computer mouse. To improve the accuracy of the eye tracker, they were encouraged to enlarge the image within comfort levels. At the completion of this self-paced analysis, they decided whether this print was of No Value, which was defined as “The impression does not contain sufficient friction ridge information to reach an identification or exclusion conclusion.” If the print was determined to be Of Value, the exemplar image was revealed and the examiner conducted a traditional comparison. Examiners were encouraged to use the computer mouse to place marks on salient points on the latent impression during the analyses stage, and this was done often. The instructions also allowed for marks to be placed on the exemplar during the comparison stage, and to link what the examiner believed to be corresponding regions, but this was rarely used. Conclusions and other determinations were communicated verbally to the subject administrator, who used a separate keyboard to enter the response, in order to not interfere with eye tracking. The experiment was double-blind, in the sense that the individuals administering the tests were unaware of whether each impression was mated or nonmated. The individuals administering the tests were not fingerprint examiners.

Examiners viewed the images on a Viewsonic VX2452mh LCD monitor at 1080p (1920x1080) resolution with 5 ms refresh running at 60 Hz from a Macintosh Mini computer. They were positioned using a chinrest 70 cm from the eye to the monitor. At this viewing distance and monitor resolution, there are 50 screen pixels per degree of viewing angle (edge to edge of the monitor was about 38°; average distance between the centers of the left and right images was about 19°).

Participants were recorded binocularly at 1000Hz using EyeLink [15] eye trackers, unless reflections from glasses allowed only monocular recording. Calibration accuracy was typically around 0.5 degrees of visual angle using 13 points of calibration and was performed at the start of each comparison. The head was stabilized with a chinrest. More details of the testing conditions are found in [12]. Appendix SI-3.1a in S3 Appendix describes our fixation segmentation and drift correction of the eye-gaze data.

Examiners terminated each comparison with one of five conclusions. They could declare No Value; identification (concluding that the two impressions came from the same finger); exclusion (concluding that the two impressions did not come from the same finger); inconclusive (meaning they were unable to reach either identification or exclusion conclusions); or after 20 minutes we allowed them to say that they required more time and we terminated the trial for sake of expediency. This final outcome was treated as Inconclusive for purposes of data analysis. Additionally, we asked whether it was a difficult comparison, using a 5-level scale from very easy to very difficult. For conclusions that were definitive, we asked examiners whether it was a borderline conclusion using the definition “If another examiner performed blind verification on this image pair and reached a different conclusion than you, how surprised would you be?”. For inconclusive responses they indicated “borderline ID” or “borderline exclusion.” Further details of the instructions are found in Appendix SI-3.2. in S3 Appendix We did not find evidence of associations between eye-gaze data and the difficulty or borderline ratings, and the behavioral results are discussed in [14].

### 2.5 Analysis data

The dataset used for our analyses consists of eye-gaze data recorded during comparison tasks collected from 121 active latent print examiners. The 52 of these examiners who completed all image pairs in the experiment completed 21 trials each (6 exemplar-exemplar and 15 latent-exemplar comparisons); overall, the 121 examiners completed a median of 5 exemplar-exemplar comparisons (mean 4.5, range 0–6), and a median of 14 latent-exemplar comparisons (mean 11.9, range 2–15). Analysis data included the fixations and conclusions from 550 exemplar-exemplar trials (243 mated, 307 nonmated, 46,042 comparison fixations), and 1444 latent-exemplar trials (804 mated, 640 nonmated, 756,671 comparison fixations). Almost all of our analyses will be on the latent-exemplar trials.

In the present study, we have the luxury of definitively knowing the “ground truth” source of every fingerprint, which is generally not true in casework. Although the concept of “missed ID” is not formally standardized, it generally is used to refer to conclusions other than identification on image pairs where identification was the consensus conclusion: inconclusive (or No Value) determinations are not considered to be missed IDs when inconclusive (or no value) is the consensus determination. For example, if a mated image pair is assigned to 20 examiners, of whom 19 are inconclusive and one concludes ID, it is not appropriate to consider the 19 inconclusive outcomes as missed IDs (even knowing the ground-truth mating): the consensus is clearly inconclusive. However, only five of the 25 mated image pairs in this dataset resulted in identification for a majority of the responses, and two mated image pairs resulted in no identification responses. Because of this limited data, we will assess the behavior associated with inconclusive outcomes on all 25 mated pairs, not limiting our analyses to non-consensus inconclusive outcomes. Thus, all inconclusive outcomes on mated image pairs are considered as missed IDs in the present work. The current study focuses on eye-gaze behavior during Comparison, and therefore trials in which the latents were described as No Value were not evaluated. A complete summary of outcomes for all image pairs is found in S2 Appendix.

### 2.6 Metric development

We undertook a series of analyses to infer the underlying cognitive and perceptual processes that are associated with each outcome, which create a set of *metrics*. Below we briefly describe these metrics and provide a complete description of each in the Supporting Information section (SI; see Appendix SI-3 in S3 Appendix). Section 2.5 defines each derived metric, and the subsequent Results and Discussion section provides the contributions of each metric to the examiners’ conclusions and outcomes. These are grouped into three categories below based on how they distinguish between candidate mechanisms. Note that because we are focused primarily on missed IDs, almost all of our analyses will be applied to mated pairs. In general, if a metric is associated with one or more of the candidate mechanisms, a discussion of this metric is included in the main text; otherwise, the metric is described in the appendices.

#### 2.6.1 Depth and completeness of the comparison: Evidence for cursory comparisons

Some missed IDs may result from an examiner prematurely terminating a comparison or failing to enter into sustained comparison-like behavior, and we developed a proxy for this sustained comparison-like behavior. In prior work on a simplified find-the-target task (i.e., locating a corresponding constellation of minutiae in a mated image) with the same participants [12], we derived a method for labeling fixations based on a variety of speed- and location-based features, categorizing the fixations into three consecutive subphases we term *scanning*, *deciding*, and *misc*. Scanning involves fast eye movements with fixations far apart and relatively little back-and-forth movement, indicating a “where is it?” period of looking for potential locations, roughly corresponding to Kundel’s “Scanning” [16]. *Deciding* generally has slow eye movement and fixations close together, often with detailed back-and-forth between the latent and exemplar images to the same location, indicating detailed work, consistent with an “am I sure?” period of deciding whether it is the correct target, roughly corresponding to Kundel’s “Decision.” Because this subphase may not actually involve a decision, this subphase is termed ‘detail’ in some contexts, and we will refer to it as such.

The derivation of subphases in [12] took advantage of knowing the location of the searched-for target. In the present work we do not know the location of the target, and so we developed a machine classifier that relies only on eye-gaze behavior that is measurable in our comparison tasks. To label each fixation, we developed features based on the eye-gaze data and associated those with the subphase on the find-the-target data. This classifier could then be used to label each fixation in the latent-exemplar comparisons without knowing the location of the searched-for target. We relied on features including the speed of movement over up to 7 consecutive fixations, the distance from the prior fixation (saccade length), detailed back-and-forth behavior between images, and time spent in each image. These features were then used by the classifier to label each fixation, and we primarily used this to filter out fixations that are not associated with a ‘detail’ behavior, under the assumption that these deciding/detail fixations provide the foundation for comparison behavior. Complete details of the machine classifier and the labeling of fixations by subphase are described in Appendix SI-3.1c in S3 Appendix.

#### 2.6.2 Measures of correspondence: Evidence for mislocalizations

**Fig 1:**
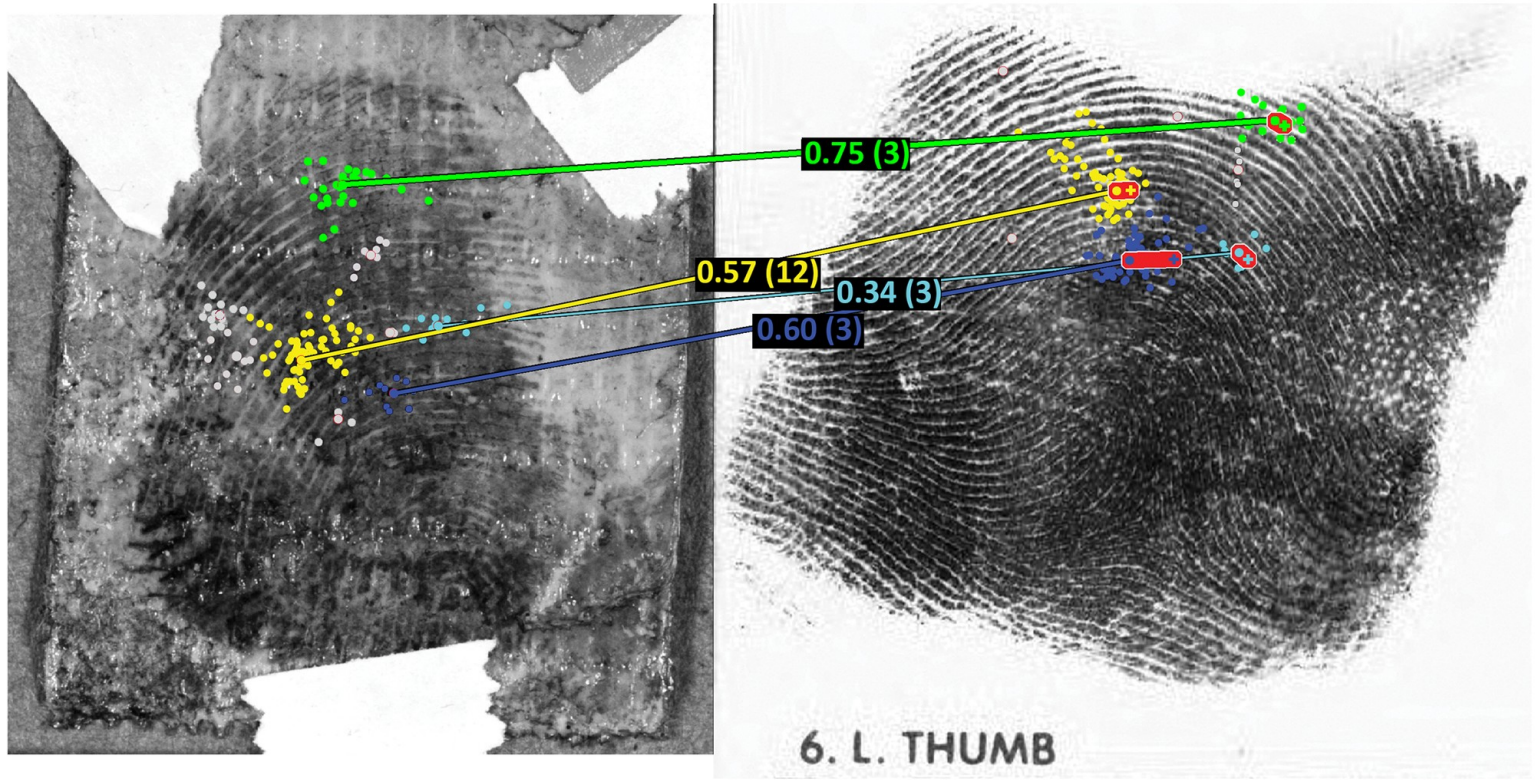
Example spatial clustering of fixations and correspondence attempts estimated by the TECA model. Small colored circles correspond to fixations, while larger circles at line endpoints correspond to the cluster centroids (regions of highest density within the cluster). Colored lines indicate estimated correspondence attempts, and the proportions indicate the strength of each correspondence attempt (see text for details). The short red lines on the exemplar indicate the distance to the projected corresponding location as described in Appendix SI-3.1e in S3 Appendix (e.g., see the short red line on the blue cluster on the exemplar). Note that only fixations from the ‘detail/deciding’ subphase are displayed and used to establish clusters, and some latent clusters are not associated if they did not have direct temporal sequences with clusters on the exemplar (and are plotted in gray). The two prints shown here are mated, and the examiner gave an identification conclusion and rated this as ‘moderately difficult’.

Latent print identification decisions require that the examiner attempt to establish correspondence between regions of two impressions. To reflect this, we developed a novel model called *TECA*, for Temporal Estimation of Correspondence Attempts. This model uses the temporal sequences embedded in the eye-gaze data from the detail fixations to identify correspondence attempts (we describe these as ‘attempts’ because nonmated pairs will not have corresponding regions, and the examiner may decide that two regions do not correspond after making a correspondence attempt). An example of the output of the TECA model is shown in Fig 1.

More information about the TECA model is found in Section 3.2 and Appendix SI-3.1e in S3 Appendix, but briefly, the set of correspondence attempts on a trial represents a summary of the collected behavior associated with looking for detail in agreement across the entire trial. To determine regions of interest on a trial, the fixations on each image are separately pre-clustered using a mean-shift algorithm, and the TECA model associates clusters on the latent impression with clusters on the exemplar impression based on temporal information.

The TECA model relies on *temporal transitions*, which are sequences in the eye-gaze record that consist of a series of fixations on the latent followed by a series on the exemplar. For each fixation, we record its cluster number, and develop a temporal transition matrix that describes the temporal associations between each cluster on the latent with each cluster on the exemplar. These temporal associations are built up over the entire trial, and then the entire matrix is normalized and correspondences are assigned based on the strength of the associations observed in the temporal transition matrix.

The TECA correspondence attempts may be equivalent to what examiners term corresponding target groups; note that each cluster generally covers more than one minutia, and as a result the correspondence attempts likely underestimates the number of corresponding minutiae. Instead, the correspondence attempts from the TECA model capture the general regions that an examiner attempted to place in correspondence. A given correspondence attempt may reflect more than a single attempt at finding detail in agreement, and the numbers in parenthesis in Fig 1 indicate the number of temporal sequences that contributed to the correspondence attempt. The percentiles in Fig 1 indicate the strength of the correspondences, which is effectively a measure of the ‘monogamy’ of the two associated clusters for each other, as described in Appendix SI-3.1e in S3 Appendix.

#### 2.6.3 Spatial metrics: Associations between regions fixated and missed IDs

We have developed four separate metrics that focus on different aspects of the spatial regions that are fixated by examiners. These provide additional support for explanations of missed IDs.

1. *Proportion of the image fixated*. For this measure, we divide the latent impression into a grid of spacing of 3 ridge widths in width and height. We consider each cell ‘visitable’ if at least three examiners placed a fixation into that cell and compute the proportion of cells each examiner visited relative to the total number of ‘visitable’ cells.
2. *Similarity to examiners with True Positive (TP) outcomes (identifications on mated image pairs)*. We used the Earth Mover algorithm to measure the similarity of fixations from each trial to the collected fixations of trials from examiners with TP outcomes on that same image pair to determine whether missed IDs were systematically different in spatial configuration from correct identifications.
3. *Spatial extent of fixations*. As a rough proxy for the thoroughness of the search, we compute the standard deviation of the fixations in the horizontal and vertical dimensions. Details are found in Appendix SI-3.1b in S3 Appendix.
4. *Proportions of fixations in low clarity areas*. For this measure, we used image-clarity markup maps [4, 17, 18] to identify whether each fixation fell in a high-, medium- or low-clarity region.

## 3 Results and discussion

Section 2.5 describes a set of metrics that we described as proxies for underlying cognitive capacities. In Section 3 we relate the metrics to outcomes by combining the mating state of each image pair (mated or nonmated) with the conclusion of each examiner (identification, inconclusive or exclusion) to create six mutually exclusive and exhaustive outcomes: True Positive (TP, identification on mated pairs), False Negative (FN, exclusion on mated pairs), True Negative (TN, exclusion on nonmated pairs), False Positive (FP, identification on nonmated pairs), inconclusive on mated pairs (IncMated), and inconclusive on nonmated pairs (IncNonMated). Trials in which the latents were described as No Value were not analyzed in most of our metrics, because there is no data for the comparison phase of the trial for No Value decisions. S2 Appendix has a summary of all outcomes for mated and nonmated image pairs.

Below we discuss various metrics and their relation to putative missed ID explanations. Additional metrics are found in the SI, including Analysis Time (Appendix SI-3.3 in S3 Appendix) and Image Clarity (Appendix SI-3.5 in S3 Appendix), neither of which showed strong evidence for associations with outcomes, but may be of theoretical or practical interest to some readers.

### 3.1 Depth and measures of completeness of the comparison: Evidence for cursory comparisons

In Section 3.1 we explore two measures that address the depth and completeness of the comparison: comparison time and the proportion of fixations associated with detailed behavior. We discuss each metric separately, and then combine them to demonstrate evidence for a particular mechanism of missed IDs: those based on *Cursory Comparisons*. A third metric, number of fixations on the latent prior to a saccade to the exemplar, was only weakly associated with outcomes (Appendix SI-3.4 in S3 Appendix).

#### 3.1.1. Comparison time

**Fig 2:**
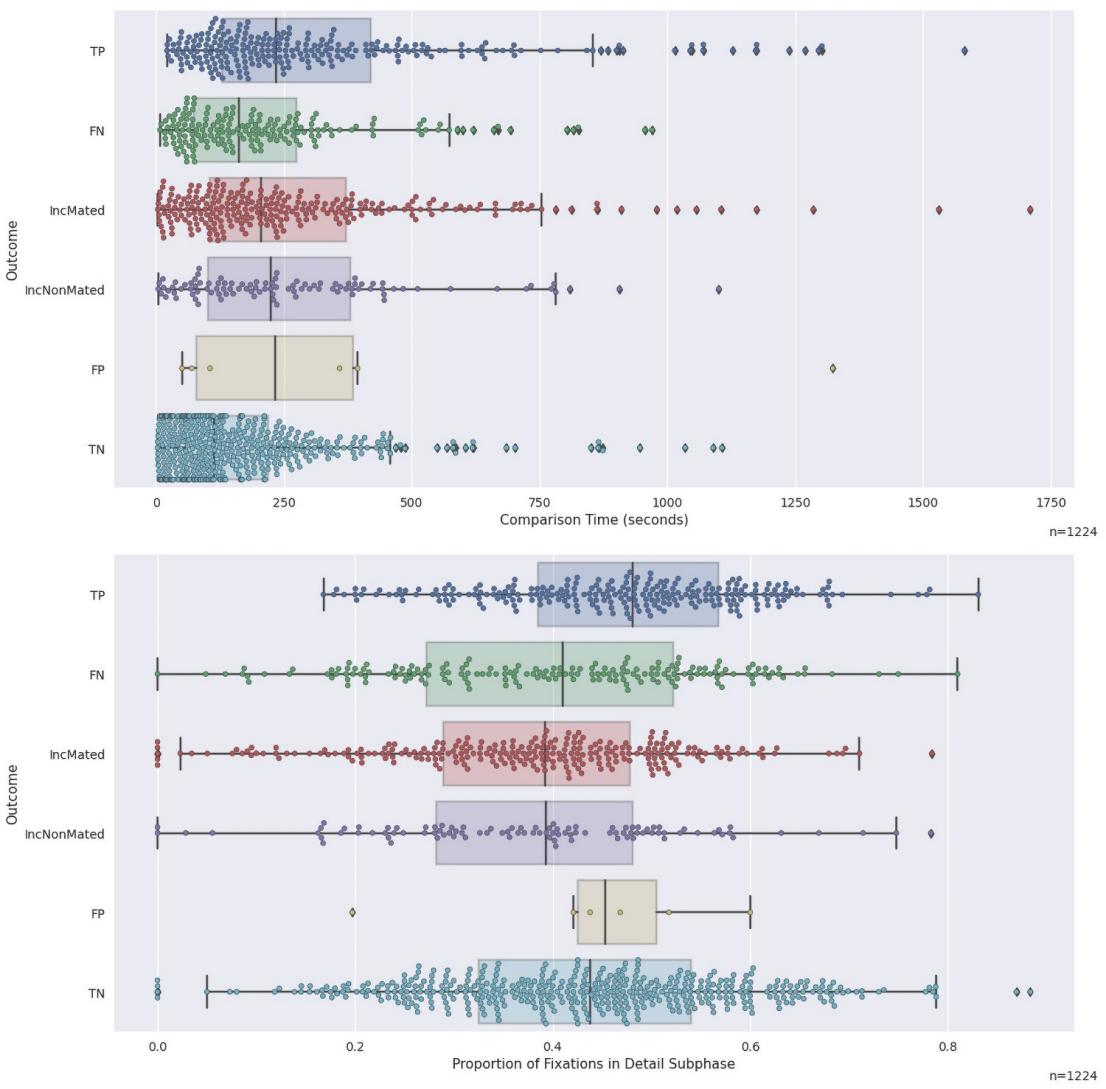
Distributions of comparison times and detail fixations with respect to outcome. Top panel: distributions of comparison times for the six outcomes. Bottom panel: distributions of proportion of fixations in the detail subphase. For all box-and-whiskers plots, the vertical line is the median, the box represents the interquartile range (25^th^ through 75^th^ percentiles), and the whiskers are 1.5 times the inter-quartile range when there are outliers outside this range (singleton diamond symbols) and express the full range of data where there are no outliers. Colored dots are data from individual trials overlaid on the boxplots. Note that there are 1444 mated trials, but only 1224 trials contribute to most of our analyses due to 220 No Value trials. For all figures, the number of trials contributing to the data is presented in the lower-right corner.

Comparison time is used as a proxy for several different cognitive capacities, including task difficulty and examiner skill. The top panel of Fig 2 illustrates the distributions of comparison time separated for different outcomes. TP outcomes tend to have longer comparison times overall than FN outcomes, and there appear to be a set of very short comparison times for FN and IncMated outcomes that we will explore below.

Is there evidence that Comparison Phase Time (or any metric) is associated with outcome? To assess whether each metric is associated with higher or lower rates of the different outcomes, we will rely on the Kolmogorov-Smirnov (KS) test [19] conducted on the distribution of the ranks of the metric. We rank each metric across all trials irrespective of outcome. For example, for Comparison Phase Time, trials in which time spent was greater than most other trials would have large percentile rank values (i.e., close to 1.0). Once ranked, if the metric were unassociated with a given outcome such as TP, trials of that given outcome would be approximately uniformly distributed among the ranks of all trials. If, for example, we find that one outcome dominates the higher ranks while another dominates the lower ranks, then this would indicate an association between that metric and outcome. We test for the statistical significance of these associations using the KS test statistic and associated p-value, although care should be taken when interpreting statistical significance because trials are not entirely independent in that there is a small set of images that are shared across multiple examiners. The KS test statistic is described in Appendix SI-3 in S3 Appendix, and uses the cumulative distribution function (CDF) to compare against the uniform distribution. As a rough guide, KS values greater than 0.1 could be viewed as meaningfully large (p-values are provided, but because of the reuse of images, may be artificially low due to the lack of independence). Although we could have conducted a traditional t-test or F-test on the raw values of each metric, we instead chose to rank the data and perform the KS test for comparison with data ranked within each image pair, as described below. We acknowledge that this ranking also tends to reduce the influence of extreme values such as very long comparison times, which might be viewed as beneficial or harmful given the goals of each analysis. To statistically separate examiner effects from image effects, we collected and ranked all trials on each image pair to create percentile scores that were relative only to trials on the same image pair. This ranking within each image pair is designed to compensate for the fact that some images contain a large area of relatively poor-clarity ridge detail, whereas others contain just a few features and the main limitation on performance is the assessment of how much specificity that detail provides. We ranked all scores on an image pair from 1 to N where N is the number of examiners who compared that image pair. By dividing these ranks by N, we establish a new set of scores that can be combined with the ranks for all image pairs.

**Fig 3:**
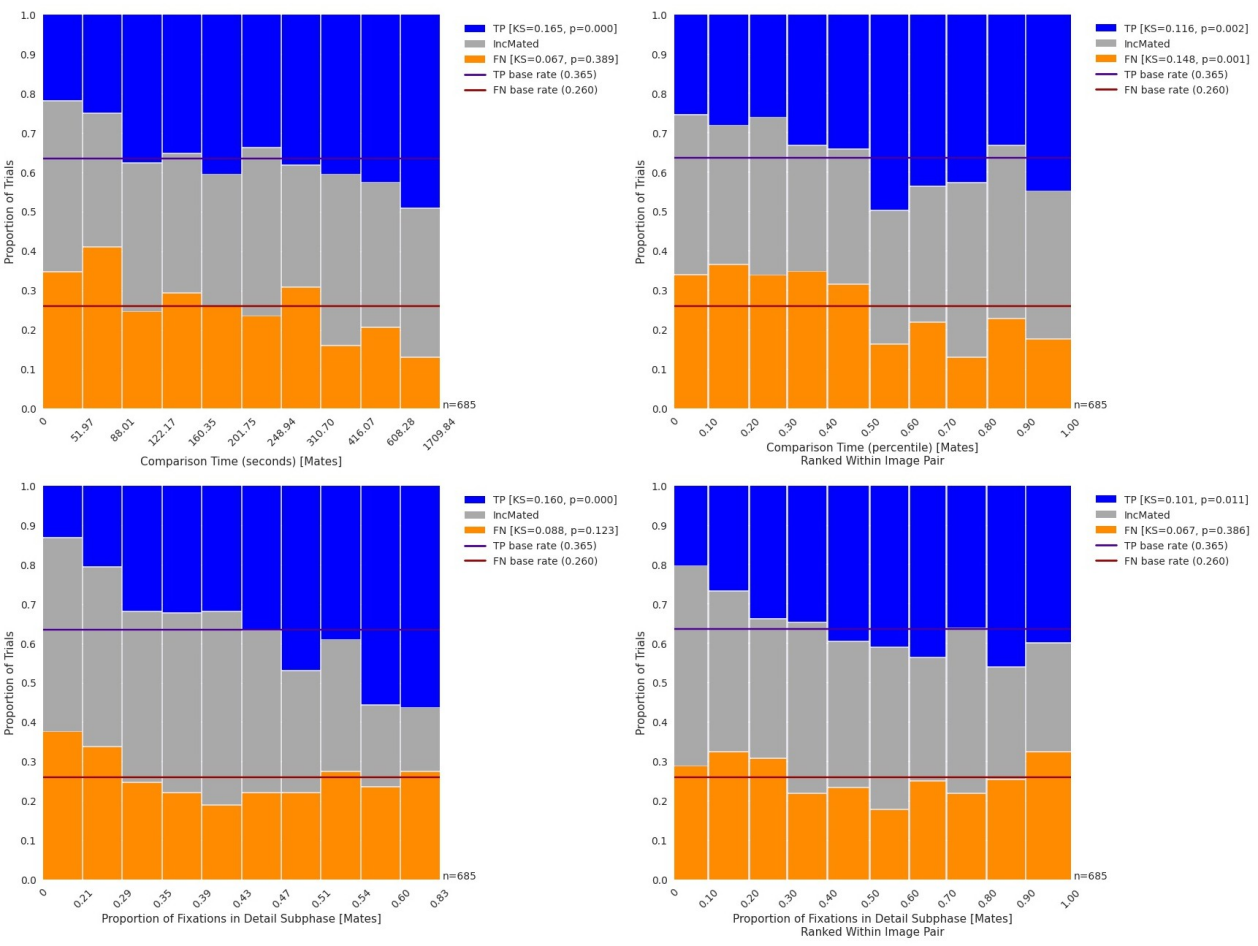
Distribution of outcomes by comparison time and proportion of detail fixations. Upper-left panel: Proportions of trials of TP, IncMated, and FN outcomes for different deciles of Comparison Time. TP outcomes tend to be associated with longer comparison times. Upper-right panel: Proportion of trials for different outcomes for comparison times that are ranked within each trial. TP outcomes tend to be associated with comparison times that are longer than most other trials on that image pair. Lower-left panel: Proportion of trials of different outcomes for Proportion of Fixations in Detail Subphase, demonstrating that TP outcomes are associated with a greater proportion of time spent in the detail subphase. Lower-right panel: Proportion of Fixations in Detail Subphase, ranked within each image pair, with weaker evidence for an association with TP.

In the upper-left panel of Fig 3, we plot the distribution of outcomes by score deciles for mated comparisons for Comparison Time. This column plot illustrates how longer comparison times are associated with more TP outcomes (KS = 0.165; *p*<0.001). We can repeat this analysis for FN outcomes, which also tests whether FN outcomes are uniformly distributed across the ranked Comparison Phase Times. There was no statistically significant association between FN outcomes and Comparison Phase Time (KS = 0.067; *p* = 0.389). After controlling for image effects (upper-right panel of Fig 3), we demonstrate a similar association between comparison time and TP outcomes (KS = 0.116, *p* = 0.002), as well as between comparison time and FN (KS = 0.148, *p* = 0.001).

*Examiners tended to take more time making identifications than making exclusions; this tendency remains even after controlling for image effects*.

#### 3.1.2. Deciding/Detail subphase

Whether an examiner enters into a sustained comparison may be an important indicator of outcome. We used the approach summarized in Section 2.5.1, and described in detail in Appendix SI-3.1c in S3 Appendix to assign each fixation to one of three subphases: scanning, detail, or misc. Of these three subphases, the detail/deciding subphase is of most interest, because it likely represents deliberative feature selection necessary for region comparison. The bottom panel of Fig 2 illustrates that TP outcomes were associated with a greater proportion of fixations in the detail subphase than FN or IncMated outcomes. The lower-left panel of Fig 3 illustrates this association for TP (KS = 0.160; *p*<0.001) and demonstrates that if a trial has few detail fixations, it is disproportionately associated with FNs and Inc. This result could be driven either by examiner effects (e.g., trials in which the examiner spent more time in the detail phase were more likely to be successful on mated pairs) or image effects (e.g., those images that are more amenable to detail processing are easier to identify), or both. To remove the influence of individual image pairs, the lower-right panel of Fig 3 graphs the distribution of this metric ranked within each image pair and demonstrates that there is weak evidence for a relation between TP outcomes and the proportion of fixations in the detail subphase (KS = 1.01; *p* = 0.011).

To put these results in context, consider the difference between the lowest and highest deciles in the lower-right panel of Fig 3. TP outcomes represent about 20% of the overall outcomes for examiners who spent the least amount of time in the detail subphase relative to their peers, while TP outcomes represent about 40% of the overall outcomes for examiners who spent the most time in the detail subphase relative to their peers. This represents a doubling of TP outcomes across the range. Thus, despite only weak evidence for an association between the proportion of detail fixations and TP outcomes, the effective increase in TP outcomes can be substantial.

*These results demonstrated an association between TP outcomes and more time spent in the detail subphase*. *IncMated outcomes tend to be associated with less time spent in the detail subphase*.

#### 3.1.3. Trials consistent with cursory comparison

In the *Cursory Comparison* mechanism for missed IDs, examiners make a decision relatively quickly or cursorily that may lead to a missed ID. We operationalize this as associated with relatively short comparison times or relatively little time spent in the detail fixation subphase. Even for long comparison time, if the examiner never enters into the detail comparison mode we assume (s)he simply could not find a reasonable starting point and is not likely to make a correct identification.

**Fig 4:**
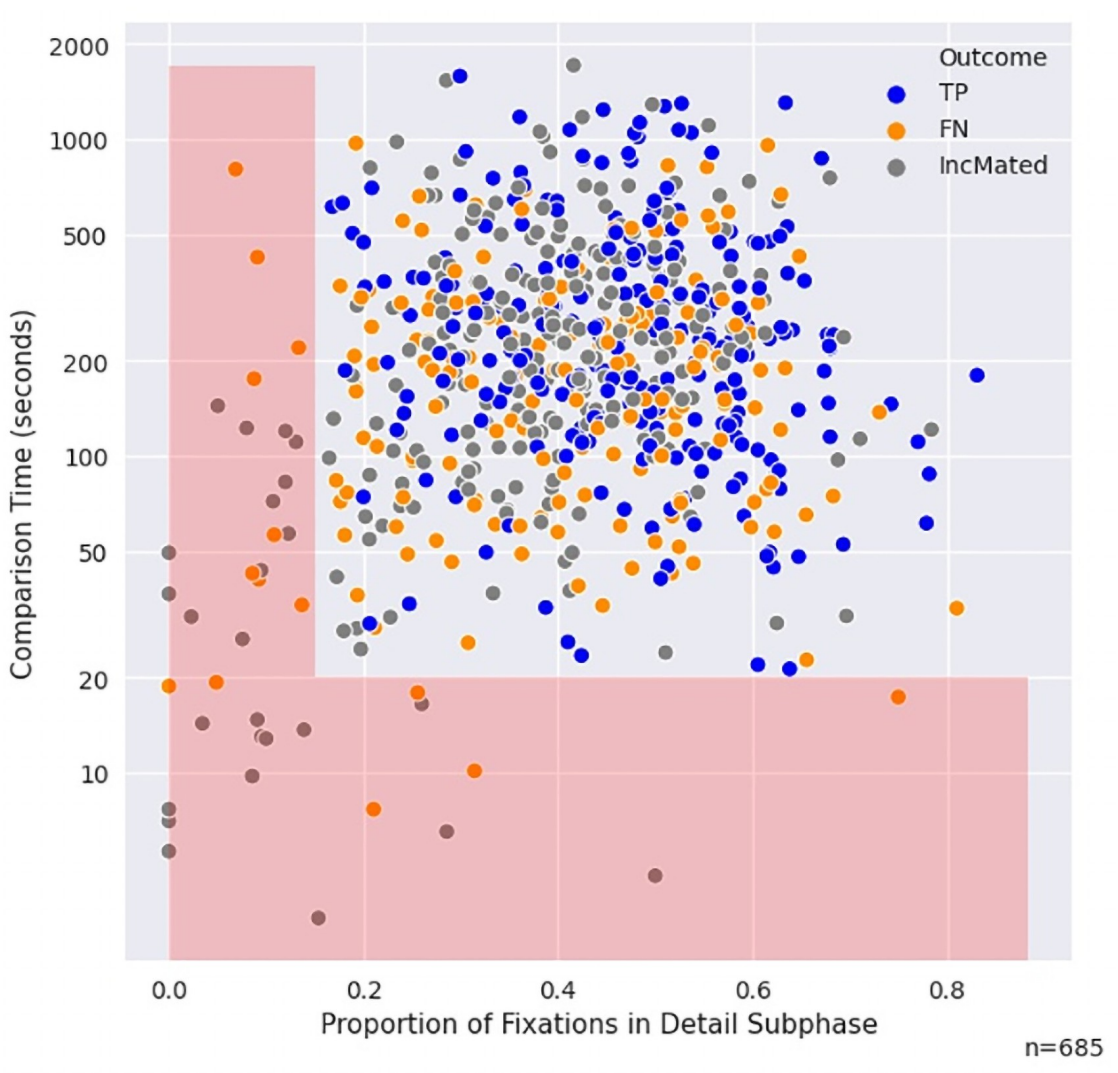
Graph of comparison time (log scale) plotted against proportion of fixations in detail subphase for mated pairs. The red area illustrates those trials that are less than 20 seconds *or* on which the percent detail is below 15% of the overall fixations. Note the complete dominance of IncMated and FN outcomes in this area.

As an illustration of which trials may be considered cursory comparisons, note that no TPs were observed that are shorter than 20 seconds *or* where examiners spend less than 15% of their time in the detail subphase (shown as the red area in Fig 4). The red area contains 5.7% of mated pairs, and 9.0% of missed IDs. Of the 39 mated trials that meet either criteria, all are missed ID trials, of which 25 are IncMated and 14 are FN. No one image pair or examiner dominates these missed IDs: of the 39 trials, there were 29 unique examiners, 15 unique image pairs, and no image pair had more than 5 trials in this category (three image pairs had 5 trials and one had 4).

*These results suggest that a proportion of missed ID trials can be described by a behavior that is consistent with a Cursory Comparison explanation*.

### 3.2 Measures of correspondence: Evidence for mislocalizations

An examiner must establish correspondences between two impressions in order to effect an identification. In this section we describe evidence for a second mechanism for missed ID that is based on mislocalization of correspondences. We will use the TECA model described previously in Section 2.5.2 and we give explicit detail of the model in Appendix S3.1e in S3 Appendix. The goal of this model is to estimate the correspondence attempts made by the examiner as she/he moves her/his eyes from the latent impression to the exemplar impression. Examples of the output of the model are found in Fig 1, and further examples are found in Appendix S3.1e in S3 Appendix.

The TECA model provides two important metrics that are proxies for the underlying belief that two areas might correspond. The first metric is the *number of correspondence attempts* as illustrated by the lines in Fig 1, and we might expect that more correspondence attempts are associated with TP outcomes. The second metric is the *accuracy of correspondence attempts*. To determine the accuracy of the correspondence attempts from the TECA model, we use the thin plate spline translation [4, 17, 18] to map the cluster center on the latent impression to a location on the exemplar impression, and then compute the distance between that point and the cluster center of the corresponding cluster on the exemplar, as shown as red lines in Fig 1. Shorter lines (i.e., smaller deviations) indicate more accurate correspondence attempts. We relate both of these metrics to outcomes in the two sections below.

#### 3.2.1. Number of correspondence attempts

**Fig 5:**
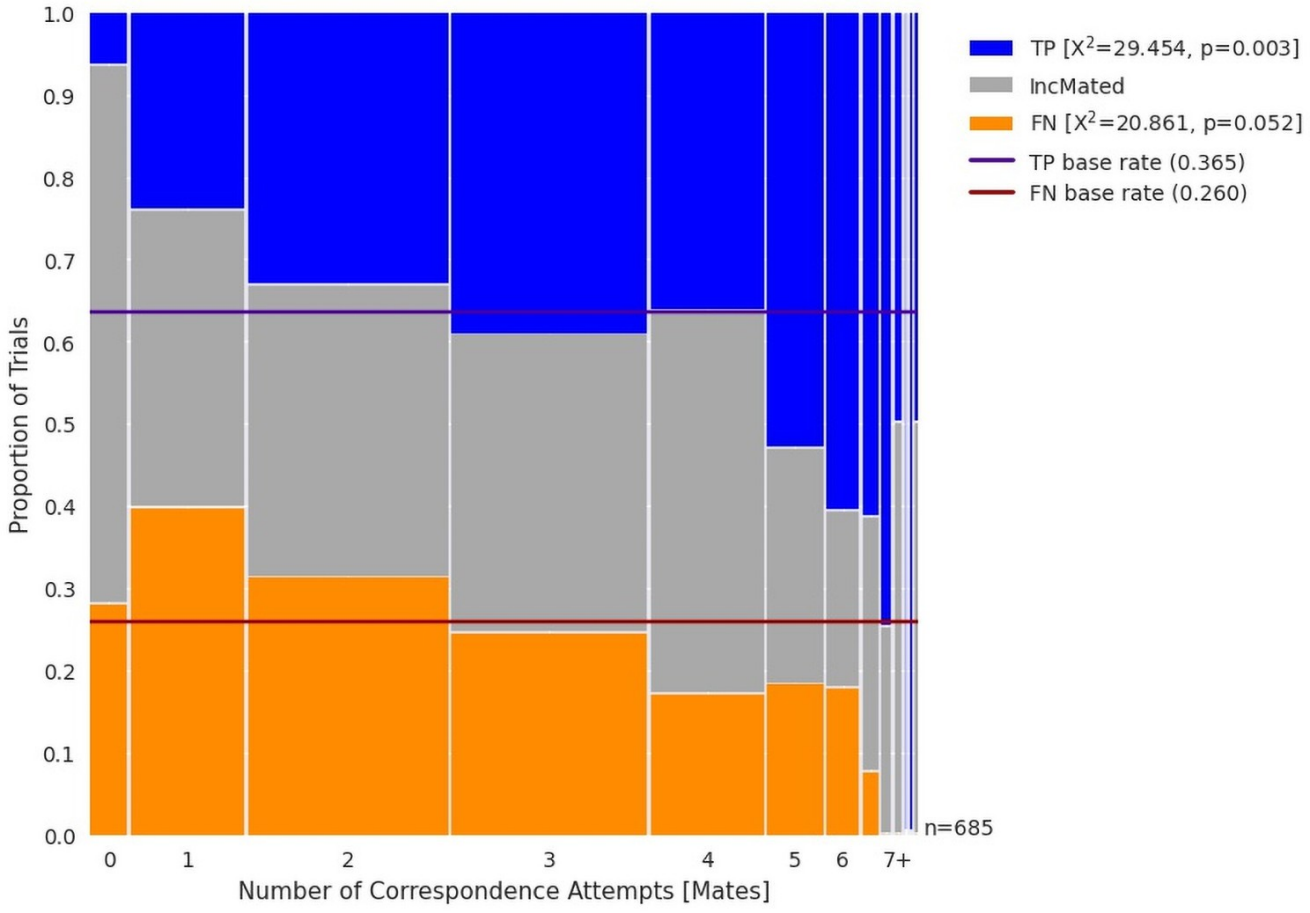
Mosaic plot of outcomes by number of correspondence attempts as estimated by the TECA model. TP outcomes are associated with a greater number of correspondence attempts.

We might expect more correspondence attempts for TP outcomes than FN trials. Fig 5 is a mosaic plot of the number of correspondences plotted against outcome. This metric differs from our other measures in that it is discrete and has relatively few values. As a result, it is not reasonable to rank our data to compute KS test statistics, nor rank within an image pair. However, we can compute the Chi-Square statistic for TP outcomes, which tests whether the distribution of TP outcomes is uniformly distributed across the Number of Correspondences. This is similar to the approach we took with the KS statistic, but is appropriate for discrete data. Fig 5 shows that TP outcomes are associated with more correspondence attempts (*X*^*2*^(12) = 29.4; p = 0.003), with more TP outcomes associated with trials with more correspondence attempts. Although FN outcomes may be associated with fewer correspondence attempts, this test did not reach our threshold for statistical significance (*X*^*2*^(12) = 20.9; p = 0.052). Note that the Chi-Square test treats the number of correspondence attempts as a nominal variable, and although we considered more metric tests such as logistic regression, we chose to use a conservative test to avoid assumptions of linearity.

*These results demonstrate an association between TP outcomes and the number of correspondence attempts*.

#### 3.2.2. Accuracy of correspondence attempts

**Fig 6:**
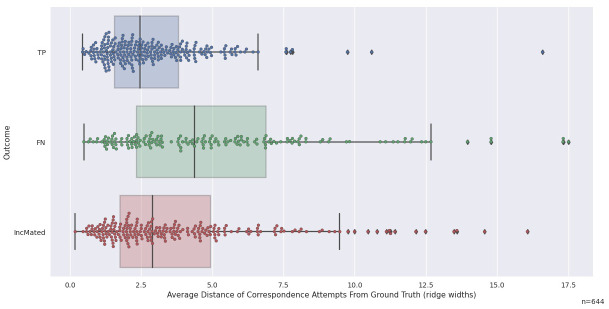
Average distance of correspondence attempts from ground truth, plotted against outcome. Only mated pairs have thin plate spline solutions that allow deviations computation. Note the large prevalence of FN and IncMated outcomes above 5 ridge widths. N = 644 because some trials have no correspondence attempts.

The *Mislocalization* mechanism suggests that correspondence attempts are more inaccurate for some missed IDs. Fig 6 illustrates the distributions of the accuracies (based on TPS projected locations) of the correspondence attempts for TP, FN, and IncMated outcomes. There is no data for non-mated pairs because they have no regions in correspondence and no thin plate spline transforms, and therefore we cannot compute the accuracy of the correspondence attempts. We compute an average for each trial across all correspondence attempts, and the distributions in Fig 6 demonstrate that some FN and IncMated outcomes have very large distance values, corresponding to very inaccurate correspondence attempts.

**Fig 7:**
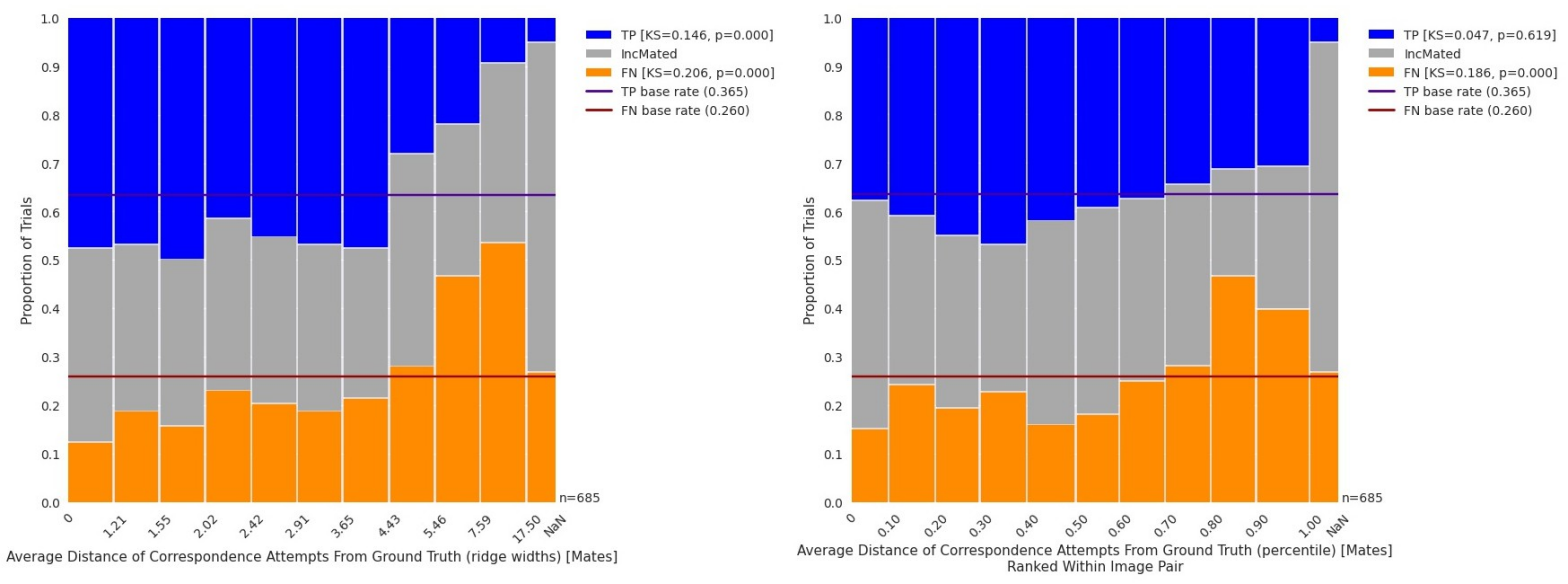
Distribution of outcomes by average distance of correspondence attempts. Left panel: Proportions of trials of TP, IncMated, and FN outcomes for different deciles of Average Distance of Correspondence Attempts. Right panel: Proportions of trials of TP, IncMated, and FN outcomes for different deciles of Average Distance of Correspondence Attempts, ranked within each image pair. NaN (not a number) values come from four mated image pairs for which we had no thin plate spline solution and could not compute deviations.

Fig 7 shows how the average distance of correspondence attempts relate to outcomes: overall rankings (left panel) and within-image-pair rankings (right panel). Greater average distances were associated with TP (KS = 0.146; *p*<0.001) and FN (KS = 0.206; *p*<0.001) outcomes. The right panel of Fig 7 demonstrates an association between the distances and outcome ranked within image pair. There was no statistically significant evidence for an association with distances ranked within image pairs and TP (KS = 0.046; *p* = .619), suggesting that much of the variation in average distances is determined by the image pairs themselves. However, we see an increase in FN outcomes at larger distances in the right panel of Fig 7, demonstrating that FN has an association with the accuracy of the correspondence attempts when ranked within image pairs (KS = 0.186; *p*<0.001).

#### 3.2.3. Trials consistent with a mislocalization mechanism

The distributions in Fig 6 have long right tails, especially for FN and IncMated outcomes, and we noted for illustration purposes a predominance of missed IDs beyond the 5-ridge-width threshold. This can also be observed as spikes in the FN data in the column plots in Fig 7, demonstrating this is not just an image effect. Trials with correspondence distances of greater than 5 ridge widths account for 22% of mated pairs, but 29% of missed IDs. Of the 151 trials that fall in this category, 126 (83%) are missed IDs. 70 (56%) of these missed IDs are FN, and 56 (44%) are IncMated.

The magnitude of this effect for FN outcomes can be observed in the left panel of Fig 7, which shows that the percent of FN outcomes for the most accurate correspondence attempts is only about 10% but approaches 50% for the least accurate correspondence attempts. When the ranks are computed within each image pair, the percent of FN outcomes is ~15% for the most accurate correspondence attempts but is ~40% for the least accurate correspondence attempts (right panel of Fig 7).

*We hypothesize that one consequence of mislocalizations is the creation of correspondence attempts (attempts by the participant to find regions of correspondence in the two impressions) that are very inaccurate*. *The subset of trials with TECA correspondences that are very inaccurate are dominated by missed IDs*. *This result suggests that mislocalization is a contributing mechanism for missed ID outcomes*.

### 3.3 Spatial metrics: Associations between regions fixated and missed IDs

The previous two sections presented evidence in support of *Cursory Comparison* and *Mislocalization* as explanations for missed IDs. In the present section, we discuss four metrics that measure different aspects of gaze behavior and assess whether they are consistent with the explanations for missed IDs as described in the Introduction. These metrics were briefly described in Section 2.5.3, and details of each are found in the SI. Image Clarity is discussed in Appendix S3.5 in S3 Appendix because the evidence for associations between Image Clarity and outcome is quite weak.

#### 3.3.1. Proportion of image visited

**Fig 8:**
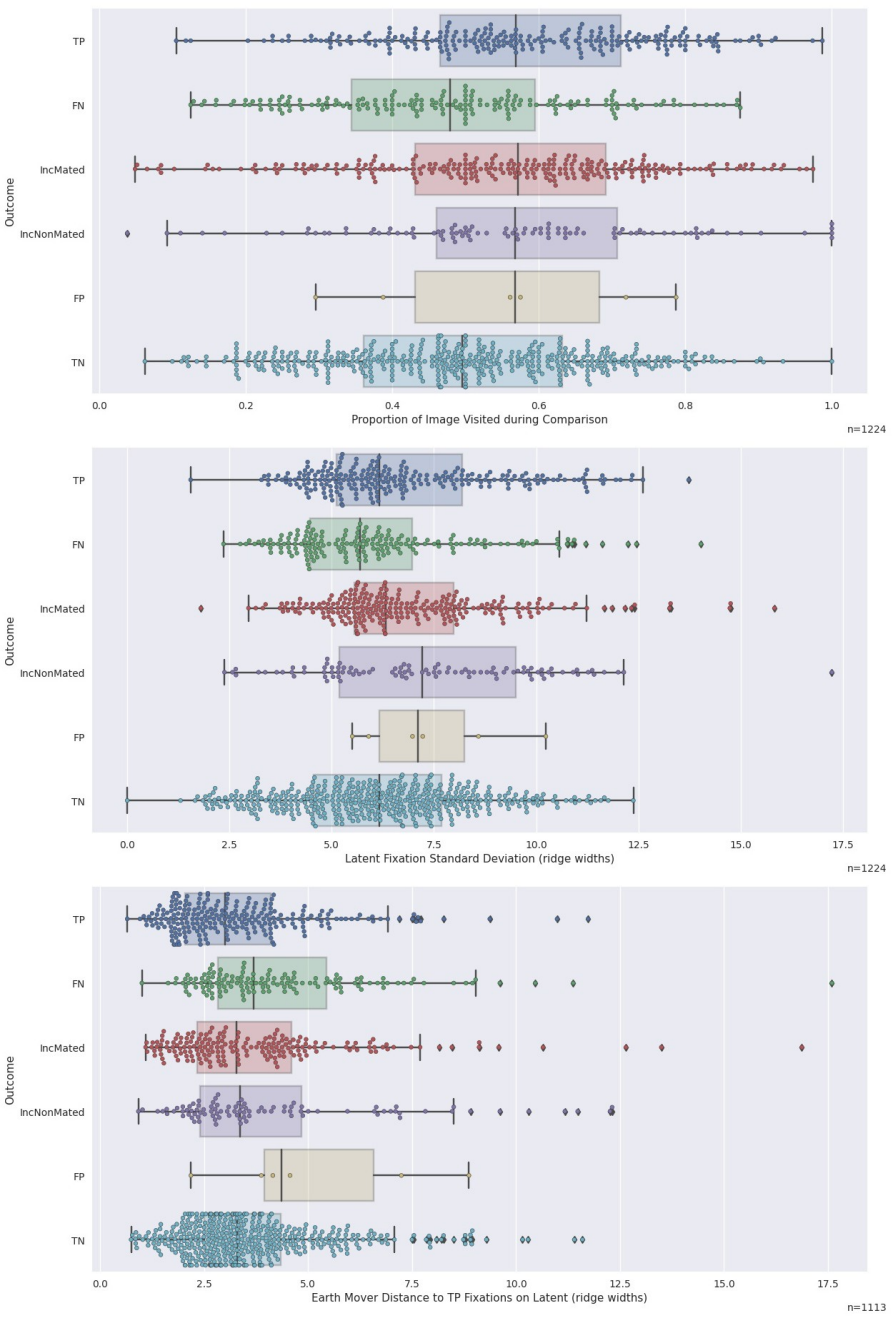
Boxplots of various metrics for each outcome. Upper panel: Proportion of Image Visited. Middle panel: Latent Fixation Standard Deviation. Lower panel: Earth Mover Distance.

**Fig 9:**
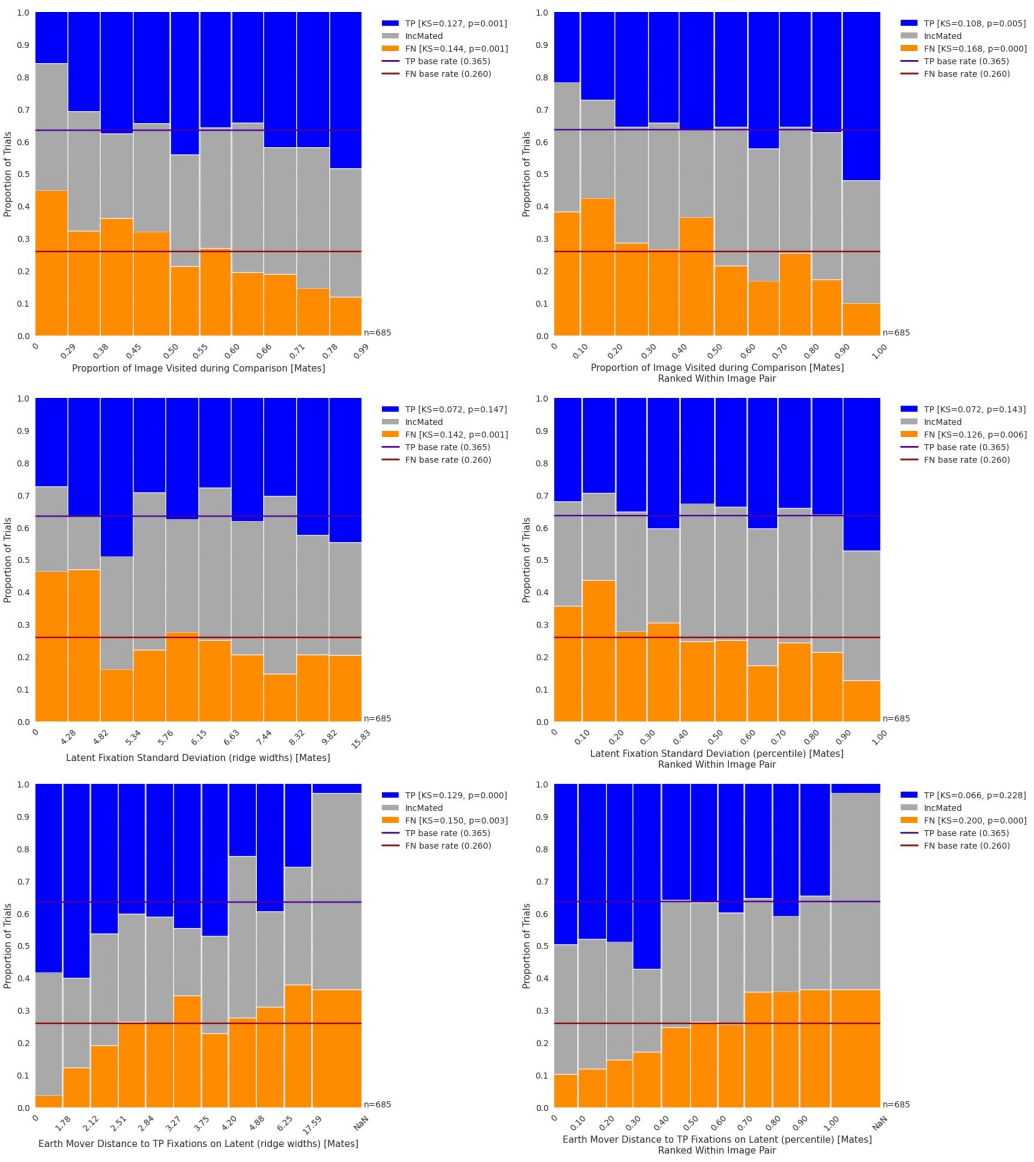
Column plots for three measures of the spatial distribution of fixations. Upper-left panel: Proportion of image visited. Upper-right panel: Proportion of image visited ranked within image pairs. Both graphs show a strong association between TP outcomes and proportion of image visited. Middle-left panel: Latent Fixation Standard Deviation. Middle-right panel: Latent fixation standard deviation, ranked within image pairs. Lower-left panel: Earth Mover Distance to TP Fixations; NaN values come from image pairs with fewer than two TP outcomes. Lower-right panel: Earth Mover Distances to TP ranked within image pair.

The top panel of Fig 8 shows the distributions of proportion of the latent image visited by examiners, separated by outcome. Recall from Section 2.5.3 that the denominator for this proportion is the number of 3-ridge-width cells that were visited by at least 3 examiners. TP outcomes tended to be associated with trials on which a higher proportion of visitable cells were visited. This trend can be seen in the top two panels of Fig 9, which demonstrates that trials on which a relatively greater proportion of the visitable area of the latent was visited tended to be associated with more TP (KS = 0.127; *p* = 0.001) and fewer FN (KS = 0.144; *p* = 0.001) outcomes. Because this is expressed as a proportion of the overall area, we would not expect strong image effects, and indeed the top-right panel of Fig 9 demonstrates a similarly strong tendency for more TP (KS = 0.108; *p* = 0.005) and fewer FN (KS = 0.168; *p*<0.001) outcomes associated with higher proportion of the image visited.

*This result suggests that visiting a greater proportion of visitable areas of the latent impression is associated with more correct identifications and fewer erroneous exclusions*. *Inconclusive outcomes do not demonstrate evidence of an association with ranks of this metric*.

#### 3.3.2. Standard deviation of latent fixations

The middle panel of Fig 8 plots the standard deviation of latent fixations (a measure of spatial dispersion that combines both the horizontal and vertical deviations; defined in Appendix S3.1b in S3 Appendix) for each trial by the six outcomes and shows that FN outcomes have smaller standard deviations than TP outcomes. The middle-left panel of Fig 9 demonstrates that this difference comes primarily from a large number of FN outcomes with very small standard deviation values. These trials largely account for a statistically significant association between this metric and FN outcomes (KS = 0.142; *p* = 0.001) but not TP outcomes (KS = 0.072; *p* = 0.147). A similar result is found for FN outcomes (KS = 0.126; *p* = 0.006) and TP outcomes (KS = 0.072; *p* = 0.143) when standard deviations are ranked within each image pair.

*The FN trials with very small standard deviations are consistent with the Cursory Comparison mechanism and suggest that on some trials examiners who reach a FN outcome explore less of the image than their peers*.

#### 3.3.3. Earth Mover distances

Examiners who come to the same conclusions might also look at similar areas of the impressions. In related work Busey, Yu [20] used a global measure of the similarity of the two constellations of fixations and found that examiners were more similar as a group than novices when constrained to have the same comparison time. They used the Earth Mover distance (EMD) [21, 22] as a measure of similarity of two sets of fixations. The EMD computes the amount of ‘work’ that is required to move one set of fixations onto another set. It has also been used in similar contexts to estimate the degree of spatial alignment of two sets of fixations [23, 24].

In the present work, we used the EMD to compute distance of each examiner’s fixations to the set of *all* fixations (across multiple examiners) who made TP outcomes on an individual pair. We calculated EMDs for fixations on the latent and exemplar impressions separately, but the results were similar. We might expect smaller EMDs for a set of fixations from a TP outcome when compared with the collection of all TP fixations, as opposed to fixations from a FN outcome when compared to the collection of all TP fixations. This finding would suggest that correct examiners are all correct in the same way, while incorrect examiners are more variable. To avoid dependencies, the current examiner is removed from the pool of correct examiners when computing the Earth Mover Distance for that examiner. We require at least two TP outcomes for an image pair in order to compute the EMD.

The bottom panel of Fig 8 illustrates that TP outcomes have smaller EMDs (and therefore higher similarity to other TP outcomes) than FN outcomes. The bottom panels of Fig 9 illustrate these data in column form and reveal an association between Earth Mover Distances and TP (KS = 0.129; *p*<0.001), as well as FN (KS = 0.144, *p*<0.001). When ranked within image pair, there is no statistically significant evidence for an association between EMD and TP outcomes (KS = 0.066, *p* = 0.228) but the association with FN is still present (KS = 0.200, *p*<0.001).

*As measured by EMD*, *the locations where examiners fixate tend to be more homogenous among TP outcomes than between TP and FN outcomes*. *FN outcomes may be affected by the same Mislocalization mechanism discussed in Section 3*.*2*, *making their fixations more dissimilar to fixations from TP outcomes*.

#### 3.3.4. Mixed effects modeling of metrics

In the analyses of the metrics described in Section 3.3, we attempted to account for image effects by ranking the metric values within each image pair. However, this analysis does not account for examiner effects: some examiners might be slower in general, and also produce more missed IDs. We addressed this in the *Cursory Comparison* analysis by ensuring that the trials that fell into the cursory comparison designation were not due to just a few examiners. However, to address this for more metrics, we constructed Mixed Effects models [25] analogous to the KS statistics. The goal of Mixed Effects modeling is to account for image effects and examiner effects as random effects in the model, while addressing the possible association between each metric and TP/FN outcomes as fixed effects.

For this analysis, we used a generalized linear mixed model [26] that included random intercepts for each Image Pair and Examiner, and included a final binomial transformation to account for our categorical TP/FN predicted outcome. We fit this model to the determine the impact each metric has on the binary response of TP vs FN. We excluded IncMated outcomes for this analysis to focus on the extreme outcomes. The metrics described in Section 3.3 tend to be correlated, and so for consistency with the KS analyses, we conducted individual models for each metric. However, we comment on a combined model below. Note that the Mixed Effects model is linear, and as such, it has the potential to miss non-monotonic effects that might arise if, say, both very slow and very fast examiners produce more FN outcomes. However, it does allow for comparisons across random and fixed effects as discussed below.

Table 1 below shows the results of individual Mixed Effects models for metrics that we considered to be of particular interest. The Examiner and Image Pair columns provide the standard deviation of the random effects associated with examiners and image pairs respectively. The Fixed Effect (slope) column provides the estimate of the fixed effect for each metric, and the subsequent columns represent the 95% confidence interval bounds around this slope estimate. Confidence intervals that do not include zero demonstrate evidence of an association between that metric and TP/FN outcomes. As can be seen in the table, most metrics demonstrate evidence for an association with outcome, which is consistent with the KS analyses described in Section 3.3.

**Table 1:** Fixed effect values and 95% confidence intervals (C.I.) for selected metrics of interest.

Metric	Examiner (Intercept) Std. Dev.	Image Pair (Intercept) Std. Dev.	Fixed Effect (slope)	Lower 95% C.I.	Upper 95% C.I.
Analysis Seconds	1.84	2.61	0.42	0.00	0.85
Comparison seconds	2.03	3.04	1.48	0.83	2.12
Earth Mover Distance to TP Fixations on Latent	1.37	2.05	-1.20	-1.71	-0.69
Proportion of Fixations in Detail Subphase	1.77	2.39	0.76	0.28	1.23
Proportion of Fixations in Scanning Subphase	1.77	2.38	-0.46	-0.91	-0.01
Proportion of Image Visited	2.08	3.48	1.81	1.10	2.51
Average Distance of TECA Correspondence Attempts from Ground Truth	1.65	2.31	-1.40	-1.95	-0.85

The intercepts for the Examiner and Image Pair random effects can be compared against the Fixed Effect column in Table 1, and demonstrate that Image Pair, and to some extent Examiner, have a larger influence on outcome than the metrics from eye tracking. This is perhaps not surprising; we deliberately chose images that differed widely in terms of image quality and quantity. The variation among examiners is more surprising and contributes to the argument for rigorous proficiency testing (discussed below in Section 3.5) because the effect of Examiner is as large or larger than the effects of many of our behavioral metrics.

If we include all fixed effects into a single model (not shown in Table 1), only Proportion of Image Visited and Average Distance of TECA Correspondence Attempts from Ground Truth demonstrate clear evidence for an association with outcome. The strength of this evidence tends to change depending on what other metrics are included in the model, demonstrating the effects of the correlations between the metrics.

*The results from the logistic mixed models tends to replicate the results from the prior KS statistics and demonstrate that associations between the metrics and outcomes are not driven solely by examiner and image effects*. *However*, *the standard deviations of the random effects tend to be larger in magnitude than the fixed effects associated with each metric*.

### 3.4 Evidence for invalid discrepancies and sufficiency mechanisms

The evidence for the *Cursory Comparison* and *Mislocalization* explanations for missed IDs described in Sections 3.1 and 3.2 had clear translations for our eye-gaze metrics. However, the final two mechanisms for missed IDs described in the Introduction are more cognitive in nature and we would expect the eye-gaze behavior to resemble that of correct identification trials. We expect this because each mechanism assumes that the examiners are looking at similar places as those examiners who reach a correct decision, but are either interpreting that information differently, or failing to reach an identification conclusion. In the case of the *Sufficiency* mechanism, if an examiner is leaning toward an identification conclusion but fails to exceed the examiner’s personal threshold for sufficiency, this would result in an inconclusive decision. In the case of a FN outcome, an examiner might accumulate extensive evidence for correspondence, but find one distortion or feature that he or she believes is an unexplainable difference and therefore reaches an exclusion decision; this would be consistent with the *Invalid Discrepancies* explanation because the correspondence attempt is likely correct, but the interpretation is not.

The defining feature of both mechanisms is that for all intents and purposes, the eye-gaze data of missed ID trials resembles the eye-gaze data of examiners who make a TP outcome, yet the examiner made an inconclusive or even exclusion conclusion. Inspection of the distributions in Figs 2, 6 and 8 reveals many FN and IncMated trials that have metrics in the range of the TP data, which demonstrates that a substantial number of missed ID trials appear quite similar to TP trials. Our inference, therefore, is that the reason for many missed IDs lies in the perceptual interpretation (*Invalid Discrepancies*) or sufficiency threshold of the individual examiner. Unfortunately, our data does not directly speak to the prevalence of these mechanisms, and we admit that this argument is at best indirect. However, we view it as an important contribution to the discussion about the causes of missed IDs, and further data would be required to explicate the evidence for each mechanism. For example, determining whether an individual examiner tended to be risk averse or risk seeking might address the degree to which sufficiency plays a role on any particular trial. Alternatively, asking examiners to document the reason for their exclusion may also demonstrate the prevalence of invalid discrepancies. Both of these approaches address the more cognitive aspects of behavior that are beyond the reaches of eye tracking alone.

### 3.5 Atypical examiner: Multiple false positive outcomes on easy comparisons

Our analyses did not focus on identification conclusions on nonmated pairs (FP outcomes), primarily because they are so rare [1]. We observed only six FP outcomes on our latent-exemplar impressions. However, one examiner made two of those latent-exemplar FP outcomes, in addition to four FPs on the exemplar-exemplar images, which were included to obtain eye-gaze data on easy comparisons. In this section we discuss the eye-gaze data for this participant and demonstrate that, based on the gaze behavior, these are likely not transcription or clerical errors. Instead, they appear to be genuine attempts to perform the task. The behavioral conclusions for this examiner are also discussed in [14].

**Fig 10:**
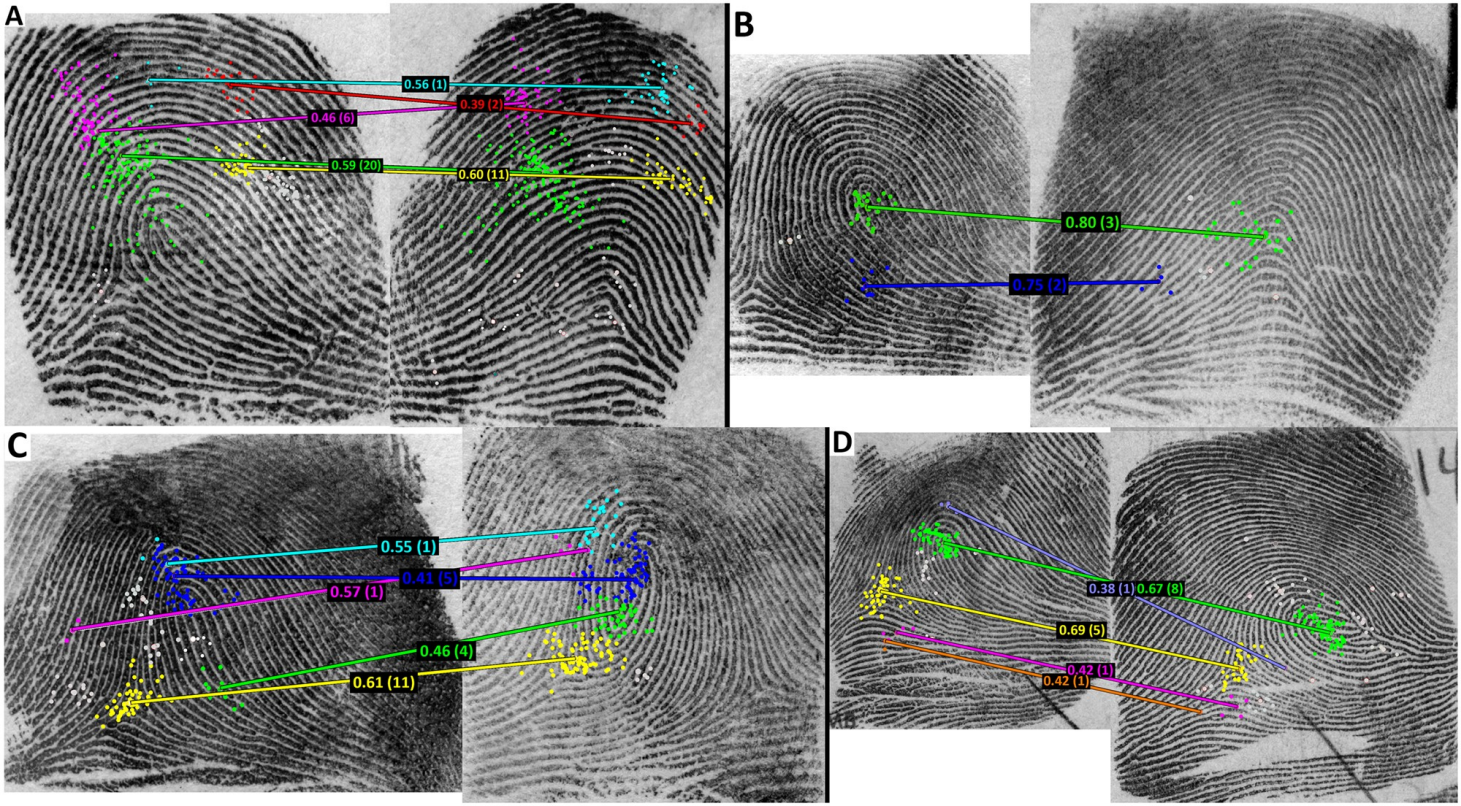
Eye-gaze data from an atypical participant who made four false positive responses on nonmated images that demonstrate high clarity in the latent impression. Each quadrant of the figure is one image pair, and colors are clustered detail fixations. Lines are the estimated correspondence attempts from the TECA model. Proportions represent the strength of the estimated correspondence attempts, and numbers in parentheses are the number of temporal sequences that associated the two linked clusters. Note that in some cases the examiner is ignoring large discrepancies, such as the delta in the right image of panel C.

Fig 10 illustrates the four examples of FP outcomes on easy (exemplar-exemplar) comparisons from this participant. Every other exemplar-exemplar trial resulted in a correct outcome (TN on nonmated image pairs and TP on mated image pairs, with no No Value, inconclusive, or erroneous determinations). Three of the four image pairs have obvious differences in pattern type between the two impressions. Note that in each case the eye-gaze behavior is consistent with typical examination, and the TECA model reported 5, 2, 5, and 5 correspondence attempts on these images, where the median number of correspondence attempts for TP outcomes was 3 correspondence attempts (see S4 Appendix for the raw gaze data on these trials for this participant). The examiner rated all four trials as moderate difficulty, while all other examiners rated them as ‘easy’ or ‘very easy’ and made correct exclusion conclusions. *Thus*, *this examiner’s eye-gaze appears to be behaving in a manner that generates correspondence attempts*. This examiner also made two FP outcomes on our latent-exemplar image pairs, and the TECA model reports 1 and 4 correspondences on these image pairs.

The examiner’s behavior is typical of an examiner comparing mated impressions: The examiner exhibited detailed fixation behavior, made numerous correspondence attempts, and spent from 110 to 572 seconds on each of these four easy trials. The areas identified by the TECA model are somewhat consistent with areas that we would expect (e.g., core compared to a core and delta compared to a delta), but the examiner also ignores key areas (see the large delta on the right image in panel C of Fig 10). This examiner seems to ignore pattern class and level 1 ridge flow on this comparison.

This examiner reported on a demographic survey that she/he was not IAI certified, worked in an unaccredited laboratory, and spent less than 50% of time doing latent comparisons. There were 5 participants in the current study who meet these criteria, 2 of whom made erroneous IDs (including the current examiner). The Institutional Review Board on human subject research that approved this research required that the participants remain anonymous, and in keeping with their requirement, all cross-references between results and identities have been destroyed. Our data collection procedures are such that we are convinced that these are not clerical errors. This participant spent notably longer on these four trials than other examiners, as revealed by the comparison times on these trials. We view this case as an argument for rigorous proficiency testing [14, 27], and also suggests that eye-gaze data could be useful for training purposes to track the progression of skill acquisition.

The dataset contains four false positive outcomes on latent-exemplar comparisons from other examiners, none of whom had more than one false positive. Although this false positive rate is higher than seen in other studies, we specifically chose the images for this study to demonstrate mechanisms that recur across examiners and image pairs. Appendix SI-4 (Fig S15) in S4 Appendix depicts all fixations for this participant for these FP outcomes.

*This examiner seems to be atypical*, *in the sense that the examiner made four serious errors on easy non-mated comparisons*, *even though the eye-gaze behavior shows evidence of reasonable correspondence attempts and deliberative eye-gaze scanning*. *This may have resulted from ignoring overall pattern class or ridge flow*, *and instead focusing on smaller regions (or individual minutiae) to place in correspondence*.

## 4 General discussion

This work presents evidence for two hypothesized mechanisms for missed IDs in fingerprint examination: *Cursory Comparison* and *Mislocalization*. An additional set of metrics differentiate between TP and missed ID outcomes: Comparison Time, Fixation Subphase labeling and TECA-based metrics provided support for candidate mechanisms of missed IDs.

We observed behavior consistent with a *Cursory Comparison* mechanism that explained at least one trial from each of almost a quarter of all examiners, demonstrating that this behavior is not isolated to a few outlier examiners. What is particularly troubling about this mechanism is that it is unclear how to reduce these missed IDs. The default advice (‘take longer, do a better search and comparison process’) would be contraindicated in labs with large backlogs. However, one outcome from the present work might be to initiate a discussion within the community on how to recognize and reduce missed IDs due to *Cursory Comparison*.

We observed behavior consistent with a *Mislocalization* mechanism that explained the behavior of a number of missed IDs. Reducing mislocalization errors may involve avoiding ‘functional fixedness’ [28], which is the tendency to continue to view objects or correspondences in the manner in which they were initially perceived. In the case of latent prints, this requires questioning initial assumptions about rotation, alignment, or skin surface. Developing a set of ‘red flags’ for impressions that are likely to produce mislocalizations is also a reasonable approach to reducing missed ID outcomes due to mislocalization.

Although the focus of the present work was on the behavioral differences in TP and missed ID outcomes, we do see eye-gaze differences between IncMated and FN outcomes. Inspection of Fig 4 reveals that the *Cursory Comparison* mechanism tends be associated with inconclusive conclusions, while the *Mislocalization* mechanism is associated with both inconclusive conclusions and erroneous exclusion (FN) errors as shown in Fig 6. The Proportion of Detail Subphase also suggests that at higher values, this metric is primarily associated with a tradeoff between TP and IncMated, which is what might be expected if outcome was sensitive to differences in sufficiency across examiners. Indeed, the behaviors associated with TP outcomes were often quite similar to those associated with IncMated outcomes, but generally quite different from those of FN outcomes. When there are differences in conclusions without notable differences in eye-gaze behavior, the differences between TP and IncMated may be driven primarily by sufficiency, whereas FN outcomes may be characterized by an interpretational error (*Invalid Discrepancies*).

The magnitude of the associations we observed can be estimated by comparing the TP proportion for the largest and smallest deciles for the ranked data. For many of our measures, the proportion of TP outcomes changes markedly across the range of the metric. In the most extreme example, *Number of Correspondences*, the proportion of TP outcomes were around 5% for zero correspondences and around 60% for five to six correspondences (see Fig 5). We see similar patterns for many of our metrics such as *Proportion of Image Visited* and *Earth Mover Distance*. Likewise, for FN outcomes, the rate of FN outcomes is around 10–15% for the most accurate correspondence attempts but rises to 40–50% for the least accurate correspondence attempts (see Fig 7) even when trials are ranked within image pair. This represents more than a doubling of the FN rate and may have operational consequences for impressions where Mislocalization might be more likely (i.e., no clear core or delta).

Our observational study does not easily allow for causal explanations of our data. However, our study does demonstrate some eye-gaze behaviors that are associated with TP and missed ID outcomes, and these results raise questions that could be addressed in future research. For example, given the association between the Proportion of Detail Fixations and TP outcomes, is detail scanning (or the underlying perceptual and cognitive mechanisms that it reflects) a teachable skill? The proportion of TP outcomes rises from 20% to ~40% across deciles for the *Proportion of Image Visited* metric—could examiners be taught how to explore more of the image? Each of these questions could be addressed in future studies with traditional experimental manipulations that address causal mechanisms.

Only some of the mechanisms for missed IDs are suitable to be analyzed through eye-gaze data, as purely cognitive mechanisms such as differences in interpretation or variation in sufficiency thresholds across examiners may also play a role. Although we provided some speculation on how the final two mechanisms for missed IDs (*Invalid Discrepancies* and *Sufficiency*) might be distinguished, eye-gaze data alone will likely not provide direct evidence.

Examiners commented that the mere act of measuring eye gaze resulted in a fair amount of introspection about where their eyes point, which could prove beneficial in training contexts. The visual system is quite constrained in terms of acuity, because only the central 3–5° of visual angle of gaze has the highest acuity and acuity falls off rapidly in the periphery. However, we are so adept at moving our gaze to an intended location that we are unaware of how constrained our perceptual system is. This has been termed the “grand illusion” of perception: “…why does it seem to us as if we are perceptually aware of the whole detailed visual field when it is quite clear that we do not attend to all that detail?” [29] The eye-gaze record is easily captured and presented for inspection by other examiners, and this has been shown to improve training and accuracy in novices [30]. Even consumer-grade eye trackers are becoming quite accurate and less expensive. We see a potential role for eye trackers in training settings, which would allow for discussion between the trainee and instructor about the eye-gaze behavior. For example, image pairs with known correspondences could be used in training, and a real time, gaze-contingent software tool could be used to automatically highlight the matching exemplar location for every region fixated on the latent. A real-time version of the TECA model could be used to summarize for the instructor those regions that the trainee attempted to place in correspondence. This is less intrusive than asking examiners to do markup, where they must interrupt their comparison behavior to place manual correspondences, although markup may also be useful in some training contexts. Such an approach would highlight the very correspondences that the trainee is attempting to learn and provide a summary for the instructor that would allow for rapid feedback and guidance.

In the Black Box study [1], 4.7% of responses on mated pairs were missed IDs (limited to mated image pairs on which the majority of conclusions were identifications). Although this may sound like a low percentage, the number of missed IDs could be potentially large given the total volume of latent print comparison performed in casework. Most laboratories do not routinely verify conclusions other than identification, and therefore missed IDs may not be detected operationally. The potential of increasing the rate of identifications that are supported by a consensus of examiners makes evaluation of missed IDs and their causes operationally important.

## Supporting information

S1 Appendix Fingerprint data description. (PDF)

S2 Appendix Distribution of outcomes for all image pairs. (PDF)

S3 Appendix Metric development. (PDF)

S4 Appendix Examples of fixation data. (PDF)

S1 File Overview and glossary. (PDF)

S2 File All SI files in one document. (PDF)

S1 References Reference citations for all SI Appendices. (PDF)
