# Supplementary material for: Characterizing missed identifications and errors in latent fingerprint comparisons using eye-tracking data
Source: PLoS One. 2021 May 24;16(5):e0251674. doi: 10.1371/journal.pone.0251674 (PMC8143401; doi:10.1371/journal.pone.0251674)
Supplement: S1 Appendix — (PDF) [file pone.0251674.s001.pdf]

## Appendix SI-1 Fingerprint data description

| | *Mating* | *Selection Group* | *Comparison sets* | *Mates* | *Nonmates* |
|---|---|---|---|---|---|
| Latent-exemplar | Mates | Low reproducibility mates (including erroneous exclusions) | 22 | 22 | - |
| | | Unanimous identification | 2 | 2 | - |
| | | Unanimous No Value or inconclusive | 1 | 1 | - |
| | Nonmates | Erroneous identification (actual) | 6 | - | 6 |
| | | Erroneous identification (potential) | 5 | - | 5 |
| | | Low reproducibility nonmates | 5 | - | 5 |
| | | Unanimous exclusion | 2 | - | 2 |
| | | Unanimous no value or inconclusive | 2 | - | 2 |
| | Total | | 45 | 25 | 20 |
| Exemplar-exemplar | Mates | Obvious ID | 8 | 8 | - |
| | Nonmates | Similar pattern class | 4 | - | 4 |
| | | Unrelated pattern class | 6 | - | 6 |
| | Total | | 18 | 8 | 10 |

Table S1. Basis for selection for the image pairs used in the study. Selection was based on responses in the earlier black box and/or white box studies [1, 2](4-40 previous responses per image pair, mean 12.9)

The experimental design balanced among competing objectives: to include a variety of image pairs, to collect conclusions from multiple examiners for every image pair, and to give each examiner image pairs with a similar distribution of difficulty and other attributes. To this end, of the 45 latent-exemplar image pairs, 15 were each assigned to 1/3 of the participants, and the remaining 30 were each assigned to 1/6 of the participants. These were assigned in blocks to control the assignment of attributes of image pairs. Fig S1 illustrates the design structure for all participants.

The study was designed assuming that examiners would generally complete 8-12 standard comparisons, and that many participants would not complete all assignments (in the end, the majority of participants completed all 15 assigned latent-exemplar comparisons: median 15, mean 12.1). The directed tasks and easy (exemplar-exemplar) comparisons were added based on the assumptions that they would be fast enough (< 1 minute each) that they would not perturb the counts of latent-exemplar comparisons, which were the main focus of the study.

The first four participants were each assigned only latent-exemplar comparisons, and none completed all assignments; feedback indicated that the unbroken series of difficult comparisons was overwhelming. All later participants received assignments in which the comparisons were grouped in this way:

- "A group" included 15 latent-exemplar image pairs and 6 exemplar-exemplar image pairs, each assigned to a third of all examiners. In the design, we planned for 40 examiners per image pair if 120 examiners participated (in the end, we had 121 examiners, and a mean of 39.6 A comparisons per participant). The three A groups (A1-A3) each contained 5 latent-exemplar comparisons and 2 exemplar-exemplar comparisons.

Each examiner was assigned to one A group, so all (e.g.) A1 comparisons were done by the same ~40 examiners, making it easier to do examiner-examiner comparisons (this was based on a lesson learned from the latent print black box study, in which random assignments made it challenging to assess examiner vs. image effects). The images were assigned to make the A groups as similar as possible in terms of difficulty: for example, each of the A groups contained one nonmated image pair that had received multiple exclusion responses, multiple No Value/inconclusive responses, and no ID responses in the White Box study.

- "B group" included 30 latent-exemplar image pairs and 18 exemplar-exemplar image pairs, each assigned to a sixth of all examiners. The six B groups (B1-B6) each contained 5 latent-exemplar comparisons and 2 exemplar-exemplar comparisons. The images were assigned to make the B groups as similar as possible in terms of difficulty.
- Directed tasks were each assigned to a third of all examiners (groups D1-D3). Each D group included 6-8 find-the-target tasks, up to 3 ridge-counting tasks, and up to 3 ridge-tracing tasks. The earlier assignments included 8 find-the-target tasks, 3 ridge-counting tasks, and 3 ridge-tracing tasks for each participant, but after the first 35 participants we decided that was excessive and dropped the directed tasks to 6 find-the-target tasks, 1 ridge-counting task, and 1 ridge-tracing task.

Each participant was randomly assigned one A group and two B groups. For each participant, the image pair in the A groups were randomized, then presented before the B groups (also randomized), so if they quit early, they still would complete the A comparisons even if some would not complete all B assignments. Directed tasks were interspersed (assigned as every even-numbered trial until all were assigned).

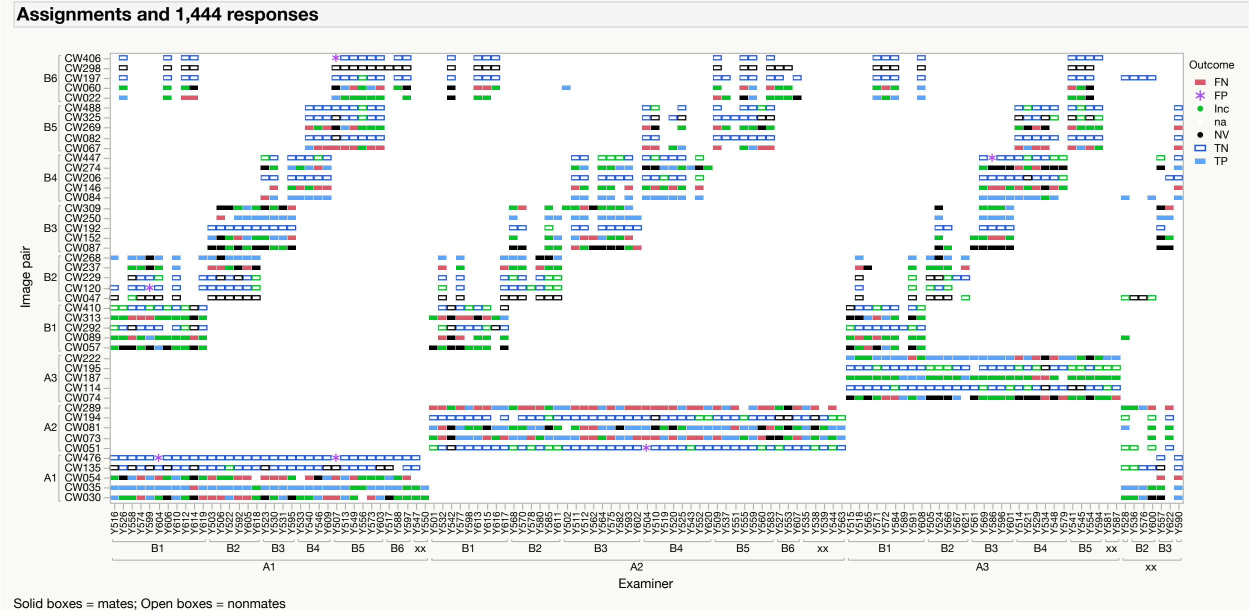


Fig S1. Design structure for the assignment of images to examiners, along with the outcome of each comparison. The images were grouped into blocks, and participants were randomly assigned to the A and B blocks. Each A image pair was assigned to 1/3 of participants, and each B image pair was assigned to 1/6 or participants. FN: False Negative (erroneous exclusion), FP: False Positive (erroneous identification), Inc (inconclusive), NV: No Value, TN: True Negative (correct exclusion), TP: True Positive (correct identification).

All images were scanned at 39.4 pixels per millimeter (1000 pixels per inch) in accordance with the prevailing standard (ANSI/NIST-ITL, 2013). Note that any references to pixel counts are integers @1000ppi, but metric equivalents are rounded.
