# Supplementary material for: Characterizing missed identifications and errors in latent fingerprint comparisons using eye-tracking data
Source: PLoS One. 2021 May 24;16(5):e0251674. doi: 10.1371/journal.pone.0251674 (PMC8143401; doi:10.1371/journal.pone.0251674)
Supplement: S2 Appendix — (PDF) [file pone.0251674.s002.pdf]

Appendix SI-2 Distribution of Outcomes for All Image Pairs

Mating	*ImagePair*	*N Rows*	*N(TP)*	*N(TN)*	*N(FP)*	*N(FN)*	*N(Inc)*	*N(NV)*
Mates	CW022	29	6	-	-	3	16	4
Mates	CW030	41	10	-	-	9	15	7
Mates	CW035	42	34	-	-	1	7	-
Mates	CW054	34	6	-	-	14	11	3
Mates	CW057	27	2	-	-	1	12	12
Mates	CW060	30	5	-	-	8	12	5
Mates	CW067	27	5	-	-	18	4	-
Mates	CW073	49	20	-	-	16	10	3
Mates	CW074	30	3	-	-	5	11	11
Mates	CW081	49	22	-	-	5	13	9
Mates	CW084	30	28	-	-	1	1	-
Mates	CW087	31	-	-	-	2	9	20
Mates	CW089	26	1	-	-	6	18	1
Mates	CW146	28	-	-	-	15	12	1
Mates	CW152	28	7	-	-	7	12	2
Mates	CW187	30	4	-	-	2	24	-
Mates	CW222	31	20	-	-	4	5	2
Mates	CW237	26	1	-	-	8	13	4
Mates	CW250	25	24	-	-	1	-	-
Mates	CW268	27	22	-	-	-	-	5
Mates	CW269	27	1	-	-	8	13	5
Mates	CW274	33	10	-	-	3	9	11
Mates	CW289	49	13	-	-	29	6	1
Mates	CW309	27	3	-	-	4	13	7
Mates	CW313	28	3	-	-	8	11	6
Nonmates	CW047	30	-	-	-	-	4	26
Nonmates	CW051	51	-	36	1	-	13	1
Nonmates	CW082	28	-	27	-	-	-	1
Nonmates	CW114	31	-	23	-	-	5	3
Nonmates	CW120	27	-	18	1	-	8	-
Nonmates	CW135	40	-	26	-	-	3	11
Nonmates	CW192	27	-	26	-	-	1	-
Nonmates	CW194	48	-	36	-	-	8	4
Nonmates	CW195	30	-	18	-	-	11	1
Nonmates	CW197	34	-	33	-	-	1	-
Nonmates	CW206	31	-	27	-	-	3	1
Nonmates	CW229	27	-	14	-	-	7	6
Nonmates	CW292	27	-	17	-	-	6	4
Nonmates	CW298	30	-	-	-	-	-	30
Nonmates	CW325	30	-	17	-	-	4	9
Nonmates	CW406	28	-	27	1	-	-	-
Nonmates	CW410	26	-	13	-	-	9	4
Nonmates	CW447	31	-	19	1	-	11	-
Nonmates	CW476	37	-	35	2	-	-	-
Nonmates	CW488	27	-	22	-	-	5	-

Table S2. Number of outcomes for each latent-exemplar image pair

Mating	*ImagePair*	*N Rows*	*N(TP)*	*N(TN)*	*N(FP)*	*N(FN)*	*N(Inc)*	*N(NV)*
Mates	CE011	47	47	-	-	-	-	-
Mates	CE012	30	30	-	-	-	-	-
Mates	CE013	26	26	-	-	-	-	-
Mates	CE014	25	25	-	-	-	-	-
Mates	CE015	25	25	-	-	-	-	-
Mates	CE016	30	30	-	-	-	-	-
Mates	CE017	29	29	-	-	-	-	-
Mates	CE018	31	31	-	-	-	-	-
Nonmates	CE001	35	-	34	1	-	-	-
Nonmates	CE002	48	-	48	-	-	-	-
Nonmates	CE003	31	-	31	-	-	-	-
Nonmates	CE004	26	-	26	-	-	-	-
Nonmates	CE005	25	-	25	-	-	-	-
Nonmates	CE006	26	-	26	-	-	-	-
Nonmates	CE007	27	-	27	-	-	-	-
Nonmates	CE008	26	-	25	1	-	-	-
Nonmates	CE009	29	-	28	1	-	-	-
Nonmates	CE010	34	-	33	1	-	-	-

Table S3. Number of outcomes for each exemplar-exemplar image pair. Dash indicates zero outcomes.

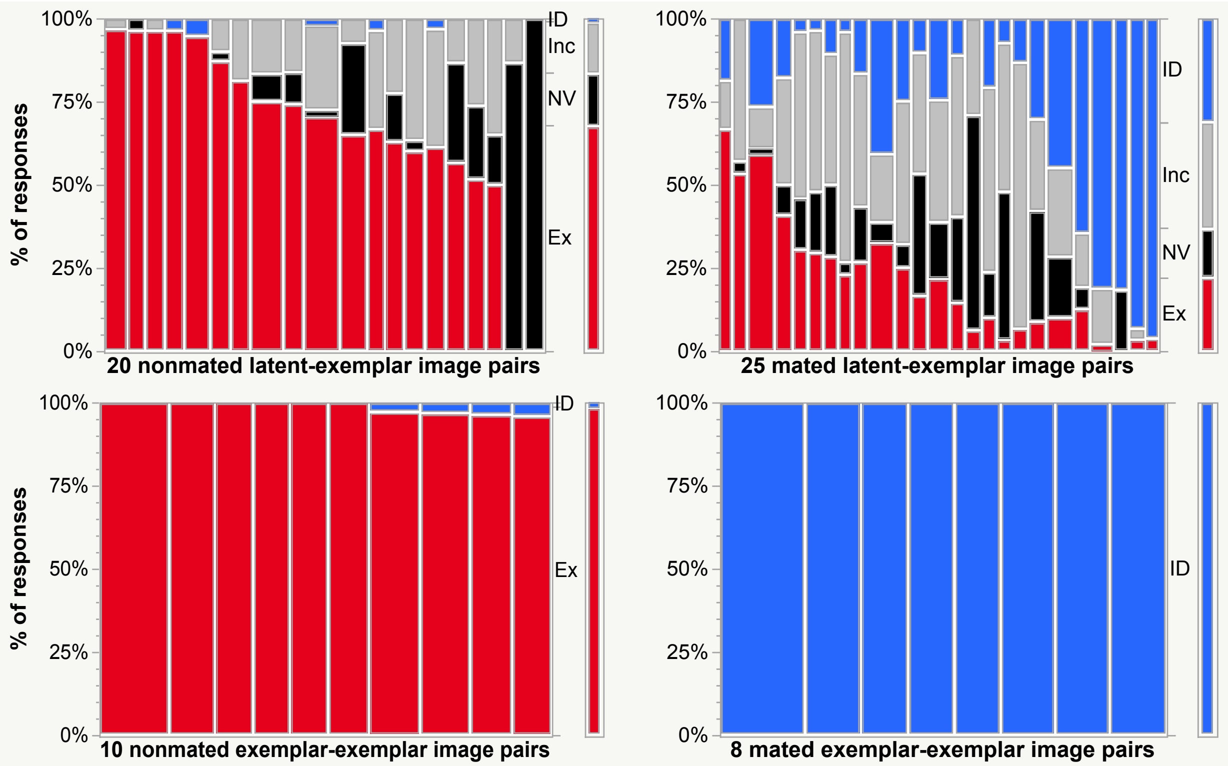

Fig S2. Distribution of conclusions for each image pair. Each column summarizes responses on one image pair, shown sorted by responses with width proportionate to the number of responses; multiple colors in a single column indicate the mix of responses by all examiners on that image pair. (n=804 mated and 640 nonmated latent-exemplar trials).

Fig S2 illustrates the distributions of conclusions for each image pair, and Tables S2 and S3 illustrate the number of outcomes for each latent-exemplar and exemplar-exemplar pair.
