# Supplementary material for: Characterizing missed identifications and errors in latent fingerprint comparisons using eye-tracking data
Source: PLoS One. 2021 May 24;16(5):e0251674. doi: 10.1371/journal.pone.0251674 (PMC8143401; doi:10.1371/journal.pone.0251674)
Supplement: S3 Appendix — (PDF) [file pone.0251674.s003.pdf]

Appendix SI-3 Metric development, test instructions, and additional analyses

Appendix SI-3.1 Metric development

Appendix SI-3.1a Fixation segmentation and drift correction

The kilohertz eye-gaze stream was segmented into fixations and saccades. We used the Engbert–Mergenthaler saccade detector [3] with a velocity threshold of 8 pixels/second (0.16°/second) and a saccade duration minimum threshold of 9 ms to identify long saccades. As in Port, Trimberger [4], a saccade required that the eye remain at rest for at least 20 ms within a ± 0.25° X-Y positional window. Further details are found in Hicklin, Ulery [5].

Drift correction was estimated and corrected using an automated procedure that used a series of 9 fixation dots that began each trial. Examiners were asked to fixate each dot, and post-processing procedures estimated the horizontal and vertical transitions of the eye-gaze data to bring the fixation in correspondence with the fiduciary points. In addition, on trials where the Analysis phase included the placement of at least four marks, we took advantage of the fact that the act of precise placement of a mark on a minutia requires extensive fixation behavior. This affords an opportunity to make small manual corrections to the vertical and horizontal placement of the points so that they align with marks placed by examiners. This procedure was done rarely and conservatively, with the goal of reducing drift while not introducing systematic bias or distortion in the fixation points.

Once we segmented the gaze stream into fixations and saccades, we computed statistics such as the proportion of fixations on the latent vs the exemplar images, the number of fixations prior to a saccade to the other impression, as well as statistics such as the spatial dispersion of the fixations. These serve as proxies for cognitive capacities such as the capacity and duration of visual working memory (as estimated by the number of fixations prior to a saccade to the other impression), and the thoroughness of the search process (as estimated by the standard deviation).

Appendix SI-3.1b Fixation spread

Fixation spread is one measure of the degree to which examiners explore the available information. We computed the combined standard deviation in the horizontal and vertical dimension for the fixations on each trial:

$$std.dev. = \sqrt{\frac{1}{n}\sum_{i=1}^{n}[(x_i - \bar{x})^2 + (y_i - \bar{y})^2]}$$

where x_i and y_i correspond to the x- and y-coordinates of the ith fixation on the image, $\bar{x}$ and $\bar{y}$ are the average x- and y- coordinates from the trial, and n is the total number of fixations on the image.

This was done separately for the latent and exemplar images, and for both the Analysis and Comparison phases. The results in Fig 8 and Fig 9 in the main paper are for the latent impressions during the Comparison phase, but results are similar on the exemplars and during the Analysis phase.

Appendix SI-3.1c Subphase determination

The goal of subphase determination is to label fixations based on our inference of the underlying cognitive behavior, with a particular focus on detailed fixation that we assume are the basis of deliberative comparison behavior. By labeling a fixation as either scanning or detail, we intend to filter the fixations to focus on behavior that is central to the comparison task.

Kundel [6] decomposed eye-gaze behavior into scanning, recognition, and deciding subphases. Hicklin, Ulery [5] adapted this approach for latent print comparisons by developing an analysis rubric based on the time taken to fixate a target feature in a simple find-the-target task. In the three subphases described in Hicklin, Ulery [5], subphase A (scanning) is the period prior to finding the target, subphase C (deciding or detailed comparison) is the period after which the examiner has found the target and has stopped considering areas outside the target, and subphase B is any time not in A or C. Subphase C involves detail-like fixation behavior associated with back-and-forth comparison of specific areas and/or studying a location in the exemplar to determine if it corresponds to the memorized fingerprint region.

That approach took advantage of the fact that the target location on the exemplar was known. However, in the present work, we do not know where the target feature is on the exemplar. An open-ended comparison (as in this study) involves a series of comparisons of target groups [7], each of which may include a series of scanning and deciding subphases Hicklin, Ulery [5] included models using logistic regression to predict subphases. Here we built on that work by using machine-learning approaches trained on the find-the-target tasks to label the analogous subphases in these naturalistic comparison trials. This classification used AdaBoostM1 with a J48 classifier, and had an 84.71% accuracy on a cross-validation set with 10 folds. Training was conducted on fixation data from [5] (4959 subphase A fixations and 9694 subphase C fixations, omitting ambiguous or subphase B fixations, on 675 trials by 117 examiners).

Once trained, this model was then used to label the fixations in the current work, using the labels *scanning* and *detail* fixations to be more readily interpretable than subphases A and C.

Several of the features used in this classification process are described as "metrics of overt behavior" in Hicklin, Ulery [5] (copied verbatim here):

- *Speed3* measures the speed of eye movement within an image in image pixels per second. *Speed3* is measured over a series of up to ± three fixations, as the sum of inter-fixation distances, divided by the time from the start of the first fixation to the end of the last fixation in the series. Fixations near left-right transitions will have fewer than ± three fixations in the series.
- We use "image visit" to refer to a consecutive series of fixations in an image (left or right), from the start of the first to the end of the last fixation in that image, bounded by the transitions between the two images.
  - TimeInImage is the duration of time spent in each image visit.
  - DetailedBackAndForth is a count of image visits in which the examiner returns to the same small area in consecutive image visits in that image. The examiner is considered to have returned to the same small area if the distance between the centers of mass of consecutive image visits in the same image is within 88 pixels (approximately 4 ridges), and the max distance between any two fixations in each image visit is no more than 176 (2*88) pixels.

In addition to these, we used two additional eye-gaze metrics:

- *DistanceOfPriorSaccade* measures the distance of the saccade preceding the current fixation (i.e., the distance from the previous fixation).
- *FrequentBackAndForth* is a count of image visits in which the examiner makes a series of saccades between the two images, spending two seconds or less in each image.

Appendix SI-3.1d Spatial clustering

Within the eye-tracking literature, images are often segmented into regions of interest. Normally this assignment of regions of interest would be done by making assumptions about the relevant distinct features in an image; for example, with faces we would assume that the eyes, nose, mouth, and ears are all regions of interest.

However, fingerprints lack such well-defined regional features, which is to say that it is difficult to draw boundaries around informative features. This is not to say that all regions are equally informative, because the distribution of fixations across the images is quite 'clumpy'. However, the boundaries between regions are not well-defined and it is unclear how one might segment visual information in a fingerprint. As an alternative, we chose to use examiner-specific regions of interest. We clustered our fixations using a mean-shift clustering algorithm [8]. We submitted all of the fixations for the latent to this algorithm to determine both a set of cluster centers, as well as those fixations that belong to each cluster; the same process was applied separately to the fixations associated with the exemplar. Note that the clustering solution uses data for only that trial; we did not combine data across examiners for this clustering solution. In addition, our use of the term 'region of interest' refers to local regions of high fixation density, not the use in fingerprint comparisons where region of interest refers to the continuous area being compared.

This algorithm has the advantage that the number of clusters does not have to be predetermined. Instead, the algorithm uses a spatial 'bandwidth' that specifies how far apart we expect two regions of interest to lie. The algorithm uses this bandwidth to cluster based on density until it reaches a region of maximum density given the spatial bandwidth parameter. An analogy would be a mountain climber who always moves up the mountain, but will move down slightly if they can see a higher point within their field of view. The bandwidth is essentially how far the climber can see, and extent of the cluster is represented by the foothills and approaches of each peak. In our application, this algorithm places cluster centers at local regions of greatest fixation density that are at least one bandwidth apart by starting at a random fixation and iteratively moving toward the point of maximum density. All of the fixations that lead to this local density maximum are labeled as associated with this cluster. We selected a bandwidth of 66 pixels, corresponding to 3 average ridge widths, (Peak-to-peak ridge distance varies by subject and is affected by distortion, but the mean in criminal databases for males is 0.022" (0.56mm) [9], so at a resolution of 1000 pixels per inch (39.37 pixels per mm), 22 pixels is a useful heuristic for ridge width) which is the only parameter for this clustering algorithm. Fig 1 in the main paper illustrates an example of clustering. Larger colored circles with red borders are cluster centers, whereas smaller colored points are the fixations associated with that cluster. Note that in many cases the clusters may have only one or two fixations and are masked by the larger symbol.

Appendix SI-3.1e Temporal transition assignment and correspondence attempt estimation: The TECA model

A foundational element of a latent print comparison task is attempts at establishing correspondences between similar regions on the two impressions (examiners often describe two regions as being 'within tolerance'). We introduce a novel measure that is intended as a proxy for this behavior. The goal is to estimate the correspondence attempts made by examiners and relate this to outcomes. Our approach will eventually assign a set of correspondences between clusters of fixations on the latent and those on the exemplar.

To address this goal, we developed a novel approach we term the TECA model, for Temporal Estimates of Correspondence Attempts. This model is inspired by work in the concept literature [10] and has the goal of jointly assigning a set of correspondences while respecting a variety of constraints. In the TECA model, we rely solely on temporal information, in that the spatial relationship of one cluster to another is done prior to estimating the temporal correspondences. We explored other approaches that include spatial information, but ultimately settled on the TECA architecture for parsimony.

Correct identification decisions may be associated with more comparison attempts relative to erroneous exclusions, and the TECA model estimates these correspondence attempts. We refer to these as 'attempts', because presumably non-mated impressions would not actually have corresponding regions, yet this behavior is still likely to be an important part of the comparison process.

Inferring correspondence attempts from the eye-gaze record is complicated by the fact that not all information is captured by an eye tracker. We know where the eyes point, but not what information is acquired, nor can we directly measure the contents of visual working memory or information acquired through the visual periphery. Examiners report placing a small area containing several minutiae into working memory (referred to as a 'target group'), making a saccade to the exemplar, and searching for a visually similar region. The relative impermanence of visual working memory requires a saccade back to the latent, typically after an average of 5-10 fixations on the exemplar. All of these behaviors are readily observed with an eye tracker.

The clustering described in Appendix SI-3.1d is done separately for the latent and exemplar images, and therefore the cluster solutions represent potential areas of interest. To establish potential correspondence links, we computed the temporal transitions between the clusters in latent and exemplar images. This relies on the supposition that the constraints of visual working memory and the need to foveate the small details in minutiae requires first

scanning for and memorizing a target group in the latent, followed by a rapid saccade to the exemplar to identify a possible correspondence. It is this temporal congruity that we wish to capture in a temporal transition.

The TECA model relies on temporal transitions from the latent image to the exemplar image. These transitions are sequences in the eye-gaze record that consist of a series of fixations on the latent followed by a series on the exemplar.

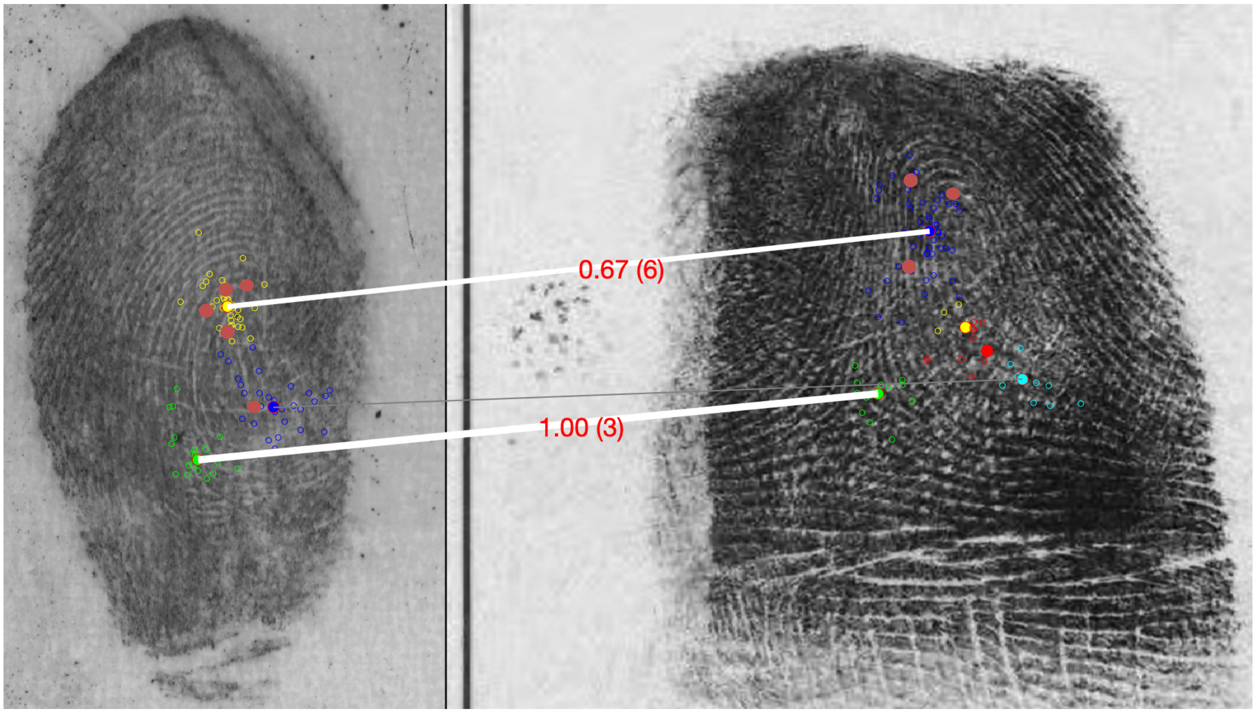

Fig S3. Illustration of a temporal transition sequence that forms the bases of the TECA model. In this hypothetical example, an examiner has made five fixations on the latent (four on the yellow cluster and one on the blue cluster, as shown by the dark red dots). The examiner then made a saccade to the exemplar, where she/he placed 3 fixations on the blue cluster. The temporal transition matrix would be updated one time for the yellow-blue pairing and one time for the blue-blue pairing. Subsequent latent-exemplar transitions will build up the entire temporal transition matrix, and there were 6 separate temporal transitions between yellow on the latent and blue on the exemplar (5 in addition the one described above). The white lines illustrate the correspondence attempts that were estimated by the TECA model, the red numbers are the relative strengths after normalization, and the numbers in parentheses are the number of temporal correspondences that contributed to each correspondence attempt. In this case, the green-green correspondence had fewer transitions (3) than the yellow-blue (6), yet had a higher relative strength after normalization because this subject was more definite when making green-green transitions (i.e., only went to green on the exemplar after fixating the green cluster on the latent).

We accumulate the identities of all of the clusters that were visited on the latent and the exemplar as part of that temporal transition. We create a *temporal transition matrix* that stores the number of times each cluster on the latent was associated with each cluster on the exemplar through a temporal transition, as shown in the example in Fig S3. Each time two clusters are associated via temporal transition, the cell of this matrix corresponding to the fixated latent and exemplar clusters is incremented by one. Note that multiple clusters could be associated during each temporal transition, because the examiner may fixate multiple clusters on the latent prior to a transition to the exemplar, and it is not clear that the last area they fixate on the latent is the feature they are searching for in the exemplar. However, our approach is designed to associate most latent clusters with a cluster on the exemplar.

Once the temporal transition matrix is populated, we use it to assign correspondence attempts between latent and exemplar clusters subject to the following constraints:

1) We first normalize the rows and columns of the temporal transition matrix by dividing each cell in a row by the row total, dividing each cell in a column by the column total, and averaging these normalized values. This normalization serves to emphasize latent-exemplar cluster pairs that were specifically looked at together. This gives fairer weight to correspondences that required few transitions and rewards 'monogamy' between two clusters. It also has the effect of establishing a weight value that ranges from 0 to 1 for all correspondences, which is important for the threshold operation described in 3) below.
2) We assign a unique correspondence for every latent cluster onto a cluster on the exemplar (unless there are more latent clusters than exemplar clusters, in which case some latent clusters will be unassigned). This assignment uses a greedy max algorithm, such that the largest cell in the normalized temporal transition matrix is assigned a correspondence, and then the rows and columns of the assigned cell are set to zero. This process is then repeated, always taking the max of the remaining entries in the temporal transition matrix to assign the next correspondence attempt.
3) Not all correspondence attempts are equally strong, and we eliminated weak correspondence attempts that fell below a threshold value of 0.3. This value was chosen based on introspection, but other values could be adopted without significantly affecting our results. The remaining high-quality correspondence attempts are

our estimates of where an examiner attempted to find 'detail in agreement' between the two impressions (to use a term favored by examiners).

To illustrate the TECA model in practice, take the following contrived temporal transition matrix (with letters arbitrarily assigned as cluster references). The numbers below represent the number of times the examiner placed a fixation in a latent cluster (A, B, or C), followed immediately by a fixation in a cluster on the exemplar (D, E, or F).

$$\begin{matrix} & A & B & C \\ D & 5 & 25 & 7 \\ E & 20 & 30 & 10 \\ F & 8 & 35 & 25 \end{matrix}$$

Each temporal transition might associate several clusters at the same time; if an examiner looked at both clusters A and B followed by a saccade to cluster D, both the A/D cell and the B/D cell would be incremented in temporal transition matrix.

The TECA model uses information from the entire matrix to disambiguate the temporal transition matrix. For example, note that cluster B is looked at often, and although it is associated with D, E, and F, none dominates. However, A is associated with E, and C with F. We can use this information to help assign a correspondence for latent cluster B, and we do this in part using normalization by dividing each cell in a column by the column total, and each cell in a row by the row total. Accordingly, the above matrix would be normalized by column:

$$X = \begin{matrix} & A & B & C \\ D & 0.152 & 0.278 & 0.167 \\ E & 0.606 & 0.333 & 0.238 \\ F & 0.242 & 0.389 & 0.595 \end{matrix}$$

and by row:

$$Y = \begin{matrix} & A & B & C \\ D & 0.135 & 0.676 & 0.189 \\ E & 0.333 & 0.500 & 0.167 \\ F & 0.118 & 0.515 & 0.368 \end{matrix}$$

and the two would be averaged elementwise to produce a final matrix, representing a score between each cluster pairing:

$$\frac{X+Y}{2} = \begin{matrix} & A & B & C \\ D & 0.143 & \mathbf{0.478} & 0.178 \\ E & \mathbf{0.470} & 0.417 & 0.202 \\ F & 0.180 & 0.452 & \mathbf{0.481} \end{matrix}$$

The greedy algorithm is used to assign the final correspondences. In this example, clusters C and F would be matched first (0.481), then B and D (0.478), and finally A and E (0.470). Since none of these values are less than 0.3, they would all be kept as correspondence attempts. Note one important detail about this assignment: Cluster B on the latent is assigned to Cluster D on the exemplar, despite the fact that it had only 25 correspondences with D in the raw temporal correspondence matrix, and 30 and 35 with the other two clusters. However, the constraints provided by the entire matrix help disambiguate individual correspondences.

Fig 1 in the main paper illustrates an example set of estimated correspondence attempts for a mated pair, and Fig S4 illustrates additional examples. We can assess the effectiveness of TECA in two ways: We can associate the number of correspondence attempts with the examiners' conclusions, and we can evaluate the correspondence attempts with respect to consensus minutia markup to identify the overall spatial accuracy of each correspondence attempt, as described next.

On most of our latent-exemplar mated pairs we have corresponding minutiae marked by multiple examiners, which were used to define consensus corresponding minutiae [11]. Based on these consensus correspondences, we used the thin plate spline (TPS) deformation model described in [12] to estimate corresponding locations between the images for all fixations. For example, using the TPS deformation model, for every fixation in a latent we estimated the corresponding location in the accompanying exemplar.

Using this TPS deformation model, we projected the region of maximum density of each latent cluster (essentially its centroid) onto the exemplar, which we treated as the "correct" corresponding location on the exemplar. We also estimate the correspondence attempt location on the exemplar using the TECA model. We compute the accuracy of each attempt by computing the distance between the TECA estimated location and the location from the TPS model on the exemplar. These distances are shown as red lines in Fig 1 in the main paper, and we might expect that TP outcomes might have smaller deviations than missed IDs.

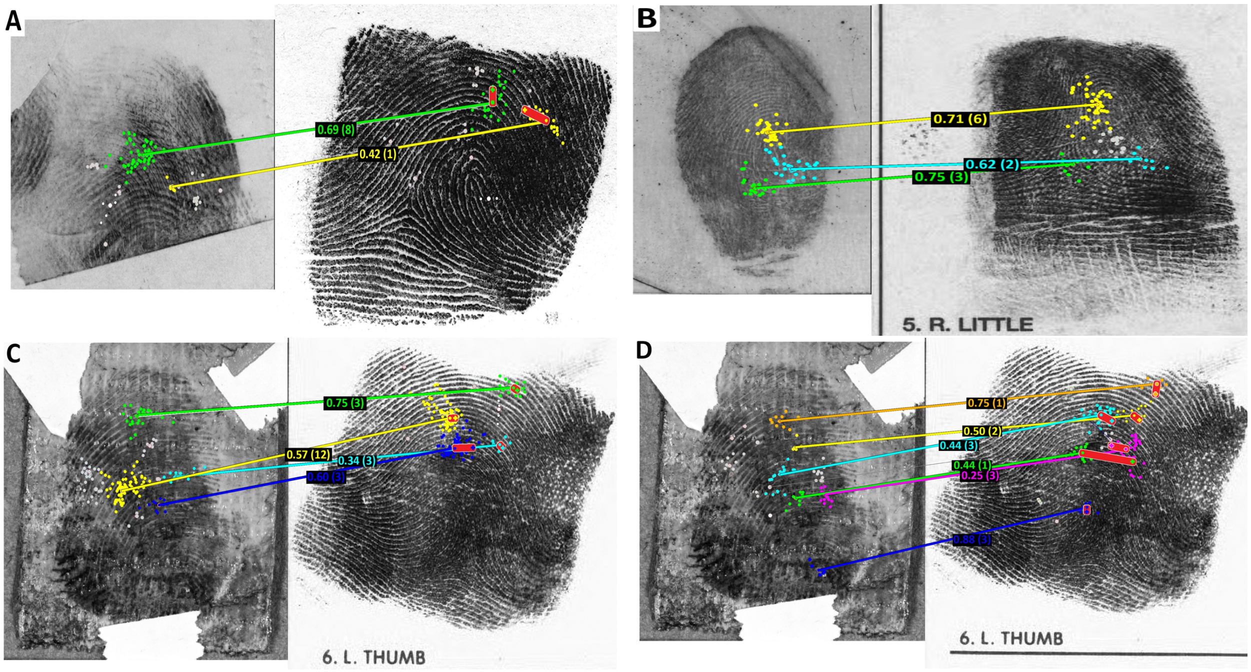

Fig S4. Additional examples of the TECA model applied to the detail fixations. Lines indicate correspondences, while red lines on exemplar images are the deviations between the estimated correspondence attempts and the ground-truth mated location of the latent cluster center. Gray lines are correspondences that were below strength threshold. Panel A: mated pair, ID conclusion, rated moderate difficulty. Panel B: nonmated pair, exclusion conclusion, rated difficult. Panel C: mated pair, ID conclusion, rated moderate difficulty. Panel D: mated pair, ID conclusion, rated very difficult.

Appendix SI-3.1f KS test statistic

Fig S5 illustrates the cumulative distribution of Comparison Time for TP outcomes, and the red vertical line is the KS test statistic. The associated p-value estimates the probability of a test statistic at least this extreme if the set of scores was sampled from the uniform distribution, which is the null hypothesis. This test is essentially asking whether a particular metric has an association with the ranks of a particular outcome, which would give a non-uniform distribution of outcomes at various levels of the metric ranks and therefore a deviation from the diagonal line. This test statistic can be computed for all of the ranks for a metric, or all ranks computed within an image pair. In either case, the collection of ranks associated with (in this case) TP outcomes is compared against the uniform distribution. Referencing the uniform distribution assumes that if there is no association, the ranks of the trials with TP outcomes

would be uniformly distributed across the range from 0 to 1. To the degree to which the TP outcomes tend to be associated with higher ranks (either for ranks computed across all trials or ranks computed within an image pair), the cumulative distribution function (CDF) will deviate from the unform distribution CDF, and as shown in Fig S5, a TP CDF below the diagonal line is associated with TP outcomes tending to be clustered at the higher ranks. As this imbalance grows, the TP CDF will deviate more from the uniform distribution CDF, leading to larger KS test statistic values.

Because of the relatively large number of hypothesis tests we present, we will use a conservative alpha of 0.01 for our threshold for statistical significance, and describe results with p-values between 0.05 and 0.01 as weak evidence for an association.

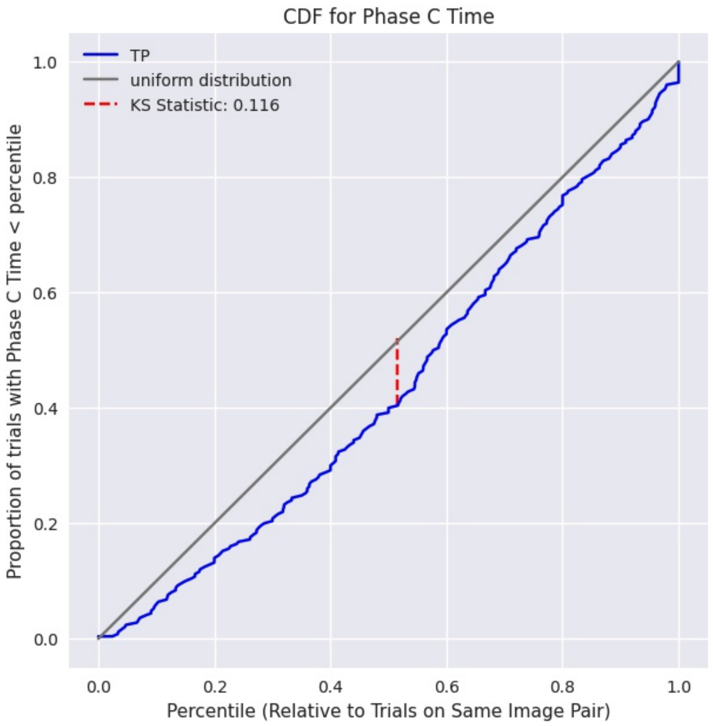

Fig S5. Illustration of the KS test statistic for the comparison of the CDF of the distribution of Ranked Comparison Time to the CDF of the uniform distribution (gray line). Ranked Comparison Time relative to trials on the same image pair is plotted for TP outcomes, and the dashed red line illustrates the maximum vertical deviation between the uniform distribution and the TP distribution. This is the KS test statistic, and it is calculated with the corresponding p-value using the Scipy package [13] to estimate the probability of obtaining a test statistic at least this extreme if the two sets of scores were sampled from the same underlying distribution. The generated graphs also used matplotlib [14], seaborn [15] and statsmodels [16].

Appendix SI-3.2 Summary of eye-tracking study test instructions

Ed. Note: this section is taken verbatim from the instructions provided to the eye-tracking study participants, but is limited to the content relevant to this paper (i.e., omitting directions regarding finding targets or following ridges).

In this study, you will be asked to perform a series of friction ridge impression examinations. Eye-tracking cameras will record the position of your head and eyes, in order to measure where on each image you are looking. Most of the test involves performing comparisons of two fingerprints. In addition, there are a few "directed tasks". It is important that you apply the same diligence that you use in casework when performing comparisons.

Analysis & Latent Value

For each examination, the software will first present a fingerprint at the left of the screen for analysis: generally, a latent, but occasionally an exemplar.

Once you complete the analysis stage, indicate the value of the print:

- *Of value for identification — The impression is of value and is appropriate for potential identification and/or exclusion if an appropriate exemplar is available.*
- *Of value for exclusion only — The impression is NOT of value for identification. The impression contains some friction ridge information that may be appropriate for exclusion if an appropriate exemplar is available.*
- *No Value — The impression does not contain sufficient friction ridge information to reach an identification or exclusion conclusion.*

Comparison/Evaluation Conclusion

At the end of comparison, you must make one of these conclusions: (say the conclusion aloud)

- *Identification — The two fingerprints originated from the same finger.*
- *Exclusion — The two fingerprints did not come from the same finger.*
- *Inconclusive — Neither identification nor exclusion is possible.*
- *Comparison not completed — You may choose this option if an examination is taking excessive time. You will be reminded of this option after 20 minutes, and asked to stop after 30 minutes.*

Borderline conclusion

Indicate whether your conclusion was a borderline decision, defined in this way:

- *If another examiner performed blind verification on this image pair and reached a different conclusion than you, how surprised would you be?*
    - Not borderline — You would be very surprised if another examiner disagreed: you would expect almost every qualified examiner to reach the same conclusion (say "NOT BORDERLINE")
    - Borderline — You would not be very surprised if another examiner disagreed: you would expect other examiners might disagree (say "BORDERLINE"). For Inconclusives, indicate "borderline ID" or "borderline exclusion."

[Why we ask: When assessing differences in eye behavior and differences in determinations, we want assistance in recognizing these borderline cases. For example, if you make an ID that is right on the edge of inconclusive, we want to be able to flag that as different from an ID you would expect every examiner to make.]

Using the software

The eye-tracking cameras that record the position of your eyes have limits on their accuracy. Because of this, we would like you to zoom in as much as you are comfortable when viewing the images.
To zoom*: move the mouse cursor over the image you want to zoom in or out. Press Z to zoom in, or X to zoom out. The zoom will be centered on the current mouse position. The two images need to be zoomed independently: you should always match the zoom level of the two prints. The two zoom levels are shown in the upper-left corner of the screen.*
To mark a minutia*: press the space bar and click on the location with the mouse.*
Discrepancies*: To indicate a minutia is a discrepancy, select the minutia and press D. The minutia will show in red.*
To link two minutiae*: select a minutia in the latent and a minutia in the exemplar and press L.*
To hide marked features*, press F.*
All other information you say aloud and the test administrator will record it.

Say "invert this image" to swap black for white in the image (tonal inversion).

Difficulty

For each comparison, say how difficult the comparison was. Routine comparisons should be indicated as "Moderate".

- *Very Easy/Obvious — The comparison determination was obvious.*
- *Easy — The comparison was easier than most latent comparisons.*
- *Moderate — The comparison was a typical latent comparison.*
- *Difficult — The comparison was more difficult than most latent comparisons.*
- *Very Difficult — The comparison was unusually difficult, involving high distortion and/or other red flags.*

Please Note

- *For the purposes of this study, "individualization" is treated as synonymous with "identification".*

- *Assume that the images provided are the only images available, and that physical evidence, lift cards, fingerprint cards, additional exemplars, and different images of these prints are not available.*
- *Every impression is a fingerprint, not a palmprint or lower joint.*
- *For an inconclusive determination, it is assumed that additional exemplars would have been requested and were not available.*
- *Make latent value determinations under the assumption that a good-quality exemplar with a large corresponding area may be available for comparison. The specific exemplar shown for comparison may or may not meet these criteria, but that should have no bearing on your value assessment.*
- *No images have already been claimed "Of Value." Some images were marked to indicate which impressions were to be scanned; such marks do not indicate that another examiner necessarily determined that the print is of value.*
- *All fingerprints are presented in an approximately upright orientation. The eye tracking software does not allow images to be rotated.*

Appendix SI-3.3 Analysis time

Fig S6 illustrates the boxplots for Analysis time broken down by different conclusions. This metric does not show a large association with different outcomes, although differences between images may mask smaller effects.

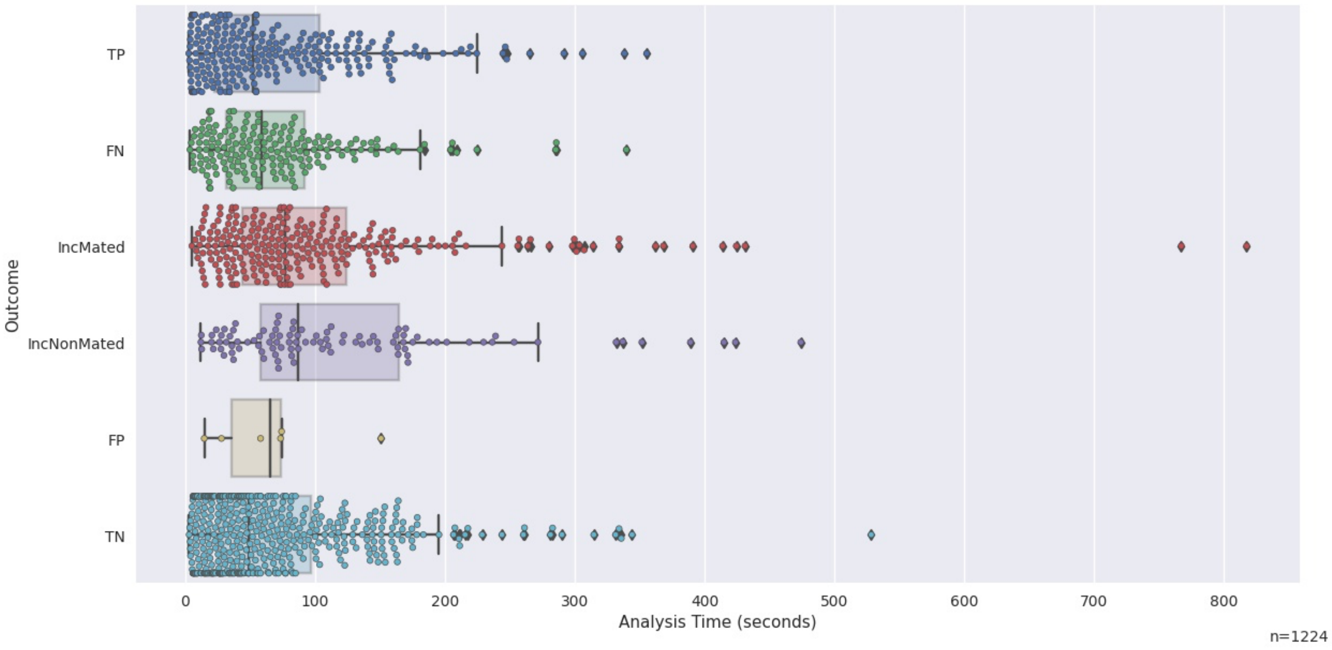

Fig S6. Distribution of Analysis Time grouped by outcome.

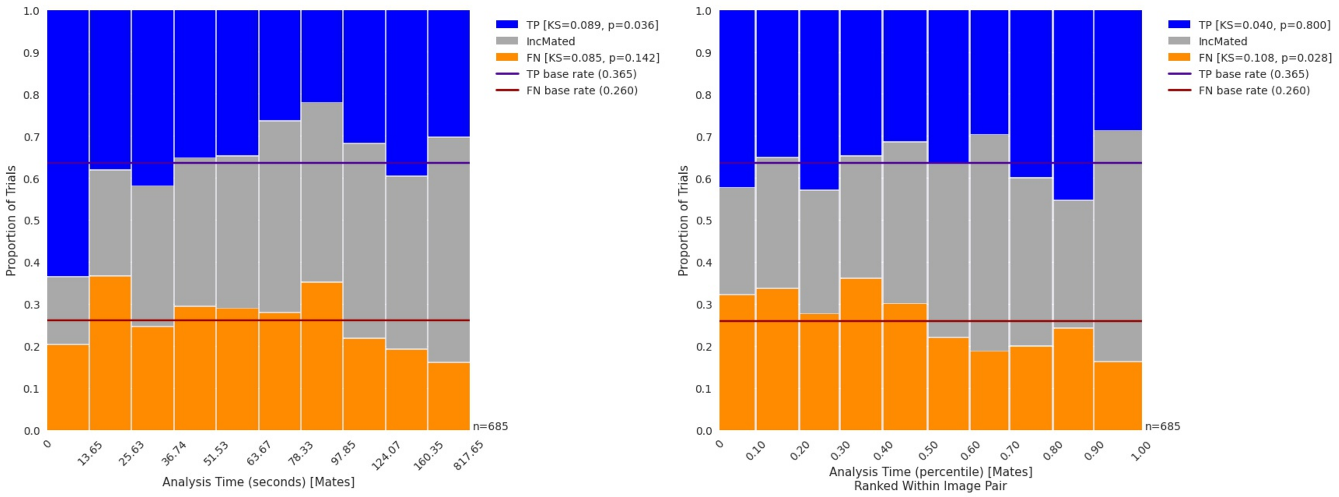

Fig S7. Distribution of different outcomes by Analysis Time, ranked by each image pair.

Fig S7. illustrates the Analysis Times ranked within each image pair. TP and TN outcomes do not show statistical support for a relation between outcome and Analysis time, with the exception of trials that had very short analysis times (left panel). These are likely due to easy images, because over half of the trials in the bottom decile in the left panel of Fig S7. are from just two images. Evidence for this association disappears once the trials are ranked by image pair (right panel of Fig S7.). FNs shows weak evidence for an association with Analysis Time.

Appendix SI-3.4 Number of fixations prior to switching images

The dynamics of eye gaze are fairly stereotyped for many tasks, not just for fingerprint comparisons: The eye tends to move (saccade) to a new position at a rate of approximately 3 times a second. However, the nature of a task and the ability of an examiner both contribute to the locations of those movements. One feature of expertise may be the ability to hold more visual features in working memory. We developed a proxy for the capacity and duration of visual working memory by counting the number of fixations on the exemplar prior to a saccade to the latent. We did not distinguish between latent and exemplar images when constructing this statistic to acknowledge the possibility that examiners may 'reverse compare' by looking for a target group on the exemplar and searching for it in the latent.

Fig S8. shows the boxplots for the number of fixations on the one image prior to a switch to the other image. TP appears to have slightly more fixations prior to a switch than FN or TN, although some images may have more regions to fixate and therefore image effects may drive this difference. These effects are modest at best, however.

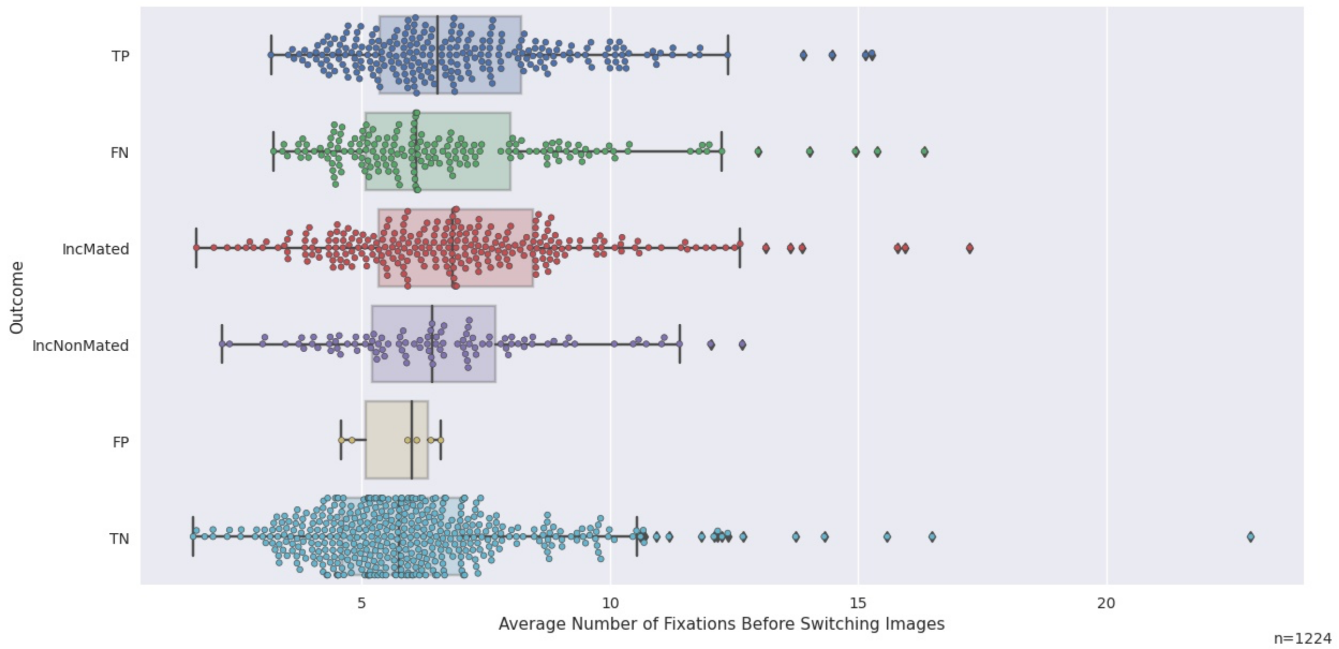

Fig S8. Average number of fixations prior to a saccade to the other image.

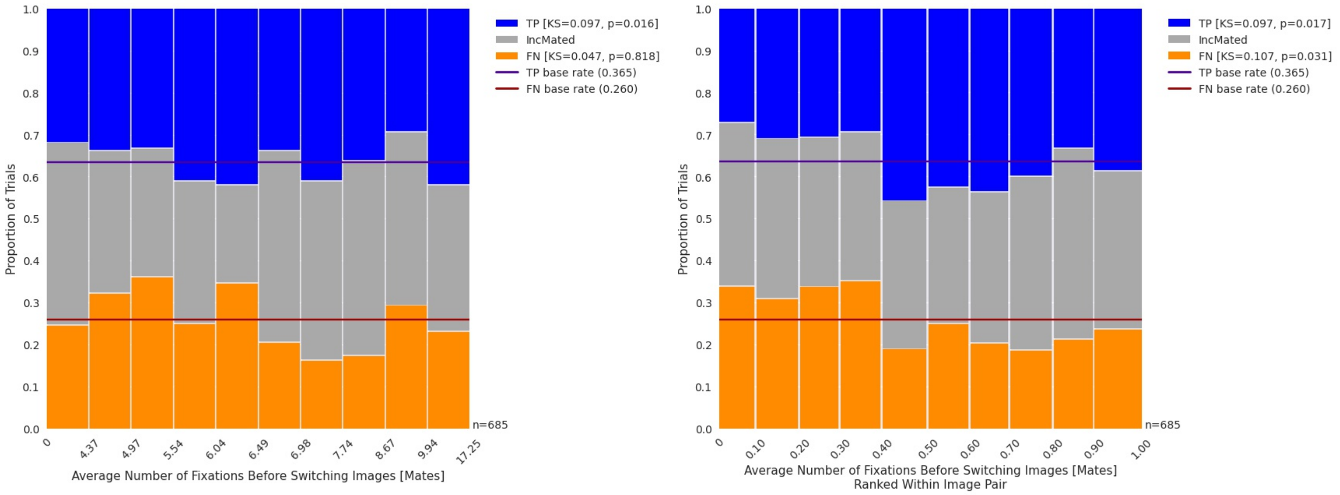

Fig S9. Distribution of different outcomes by number of fixations prior to a saccade to the other impression, ranked by each image pair.

The left panel of Fig S9 illustrates weak evidence for TP outcomes to be associated with more fixations prior to a saccade (KS = 0.097; *p*=0.016). The right panel of Fig S9 presents the histograms of this metric ranked within each image pair to reduce image effects. There is weak evidence for an association between TP and the number of fixations prior to a saccade (KS = 0.097; *p*=0.017), which does not reach statistical significance by our conservative alpha. We see a similar result between this measure and FN outcomes, with a modest tendency for more fixations to be associated with fewer FN outcomes.

The weak evidence for associations we observe may be a function of the familiarity that examiners have with the stimuli and, if we had included less experienced examiners or novices, we may have observed stronger evidence. However, this metric does not appear to be a major contributor to outcome.

Appendix SI-3.5 Image clarity

Examiners tend to describe a latent in terms of both the clarity of the print and the quantity of features present, and suggest that both play a role in the amount of information available to conduct a comparison. Examiners who are willing to look in lower-clarity regions may have different outcomes than examiners who rely primarily on higher-clarity regions. We used previously marked regions of low clarity (denoted by red and yellow in the clarity markup process) on many of our images to compute the proportion of fixations that fall in the lowest clarity regions [2].

Fig S10 illustrates that that there is perhaps weak evidence for higher numbers of fixations in low clarity regions for FN outcomes, but this could be due to image effects: Images that are of poorer clarity will lead to more fixations in poor clarity regions and also be more difficult. However, the data in Fig S10 is not consistent with the idea that better examiners are more willing to work 'in the weeds', or if so, that effect is overwhelmed by the image effects mentioned above.

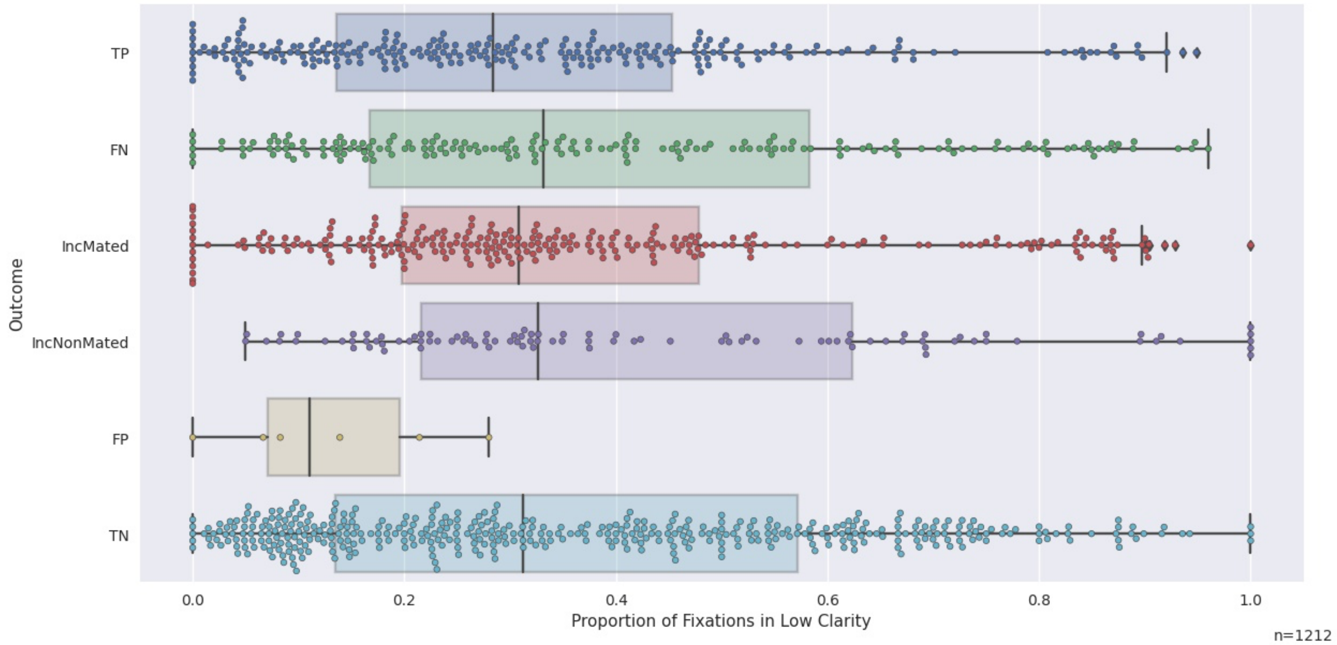

Fig S10. Boxplots of the proportion of fixations that fall in the low clarity regions of the latent impression.

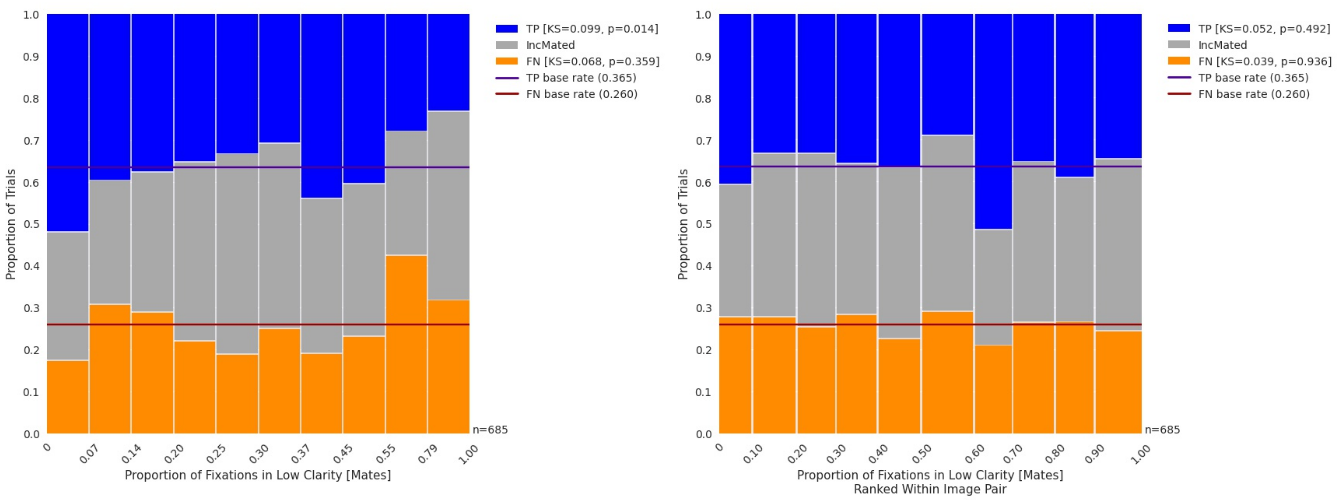

Fig S11. Relation between outcomes and the proportion fixations in low clarity regions. Left panel: data for the proportion of fixations in low clarity regions. Right panel: data the proportion of fixations in low clarity regions ranked within image pair.

The left panel of Fig S11 shows that there is weak evidence for an association between TP outcomes and the proportion of fixations in low clarity regions. In this case, TP outcomes tended to be associated with *fewer* fixations in low clarity regions (KS = 0.099; p=0.014). However, this does not mean that one should never look in low clarity areas; it could be that images that only contain low clarity areas are more difficult. Once this metric is ranked within image pairs as demonstrated by the right panel of Fig S11, there is no evidence for an association between the proportion of fixations in low clarity regions and either TP (KS = 0.052; p=0.492) or FN (KS = 0.039; p=0.936) outcomes.

These results provide weak support at best for an association between the proportion of fixations in low clarity regions and TP outcomes, which are likely driven by image effects, not differences among examiners.
