# Supplementary material for: Characterizing missed identifications and errors in latent fingerprint comparisons using eye-tracking data
Source: PLoS One. 2021 May 24;16(5):e0251674. doi: 10.1371/journal.pone.0251674 (PMC8143401; doi:10.1371/journal.pone.0251674)
Supplement: S4 Appendix — (PDF) [file pone.0251674.s004.pdf]

Appendix SI-4 Examples of fixation data

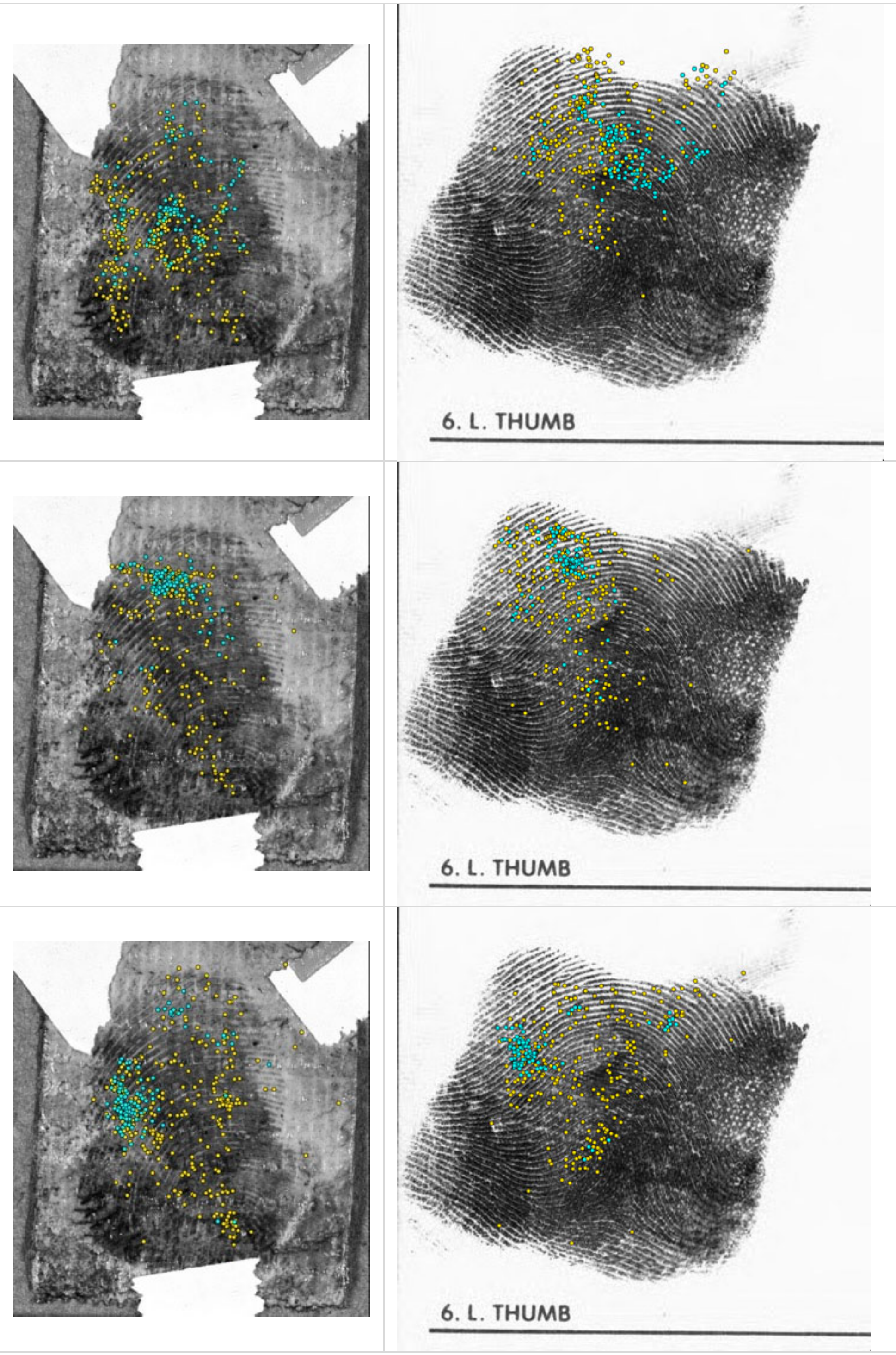

Top row:
Correct Identification
Moderate difficulty
4.9 minute comparison time

Middle row:
Erroneous Exclusion
Difficult
4.8 minute comparison time

Bottom row:
Inconclusive
Difficult
5.2 minute comparison time

Fig S12. Examples of fixations for three trials on a mated image pair (CW073). Overall conclusion counts: No Value 3, exclusion 16, inconclusive 10, identification 20, Total 49.

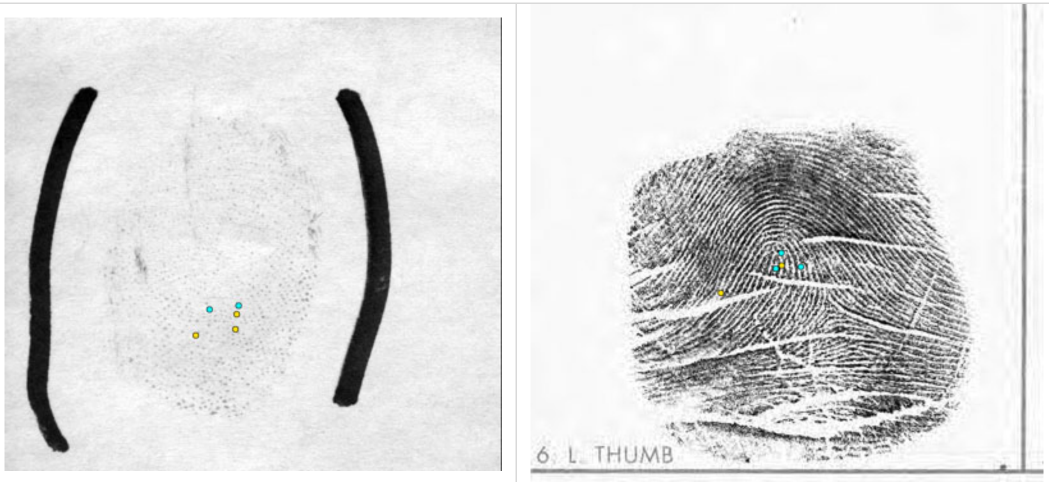	Top row: Inconclusive<br>Very Difficult<br>5 second comparison time<br>(after 1.8 minute analysis time and determination of Value for exclusion only)
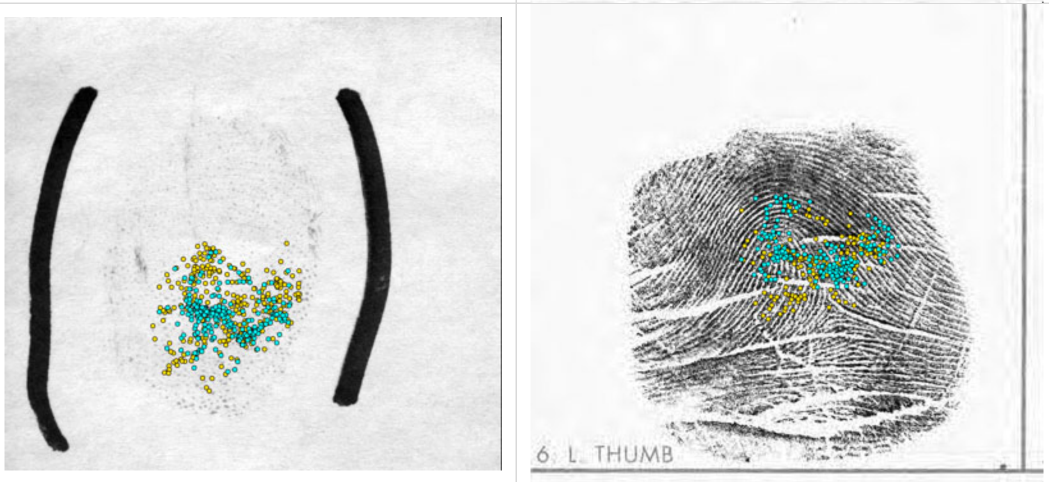	Middle row: ID<br>Very Difficult<br>7.2 minute comparison time
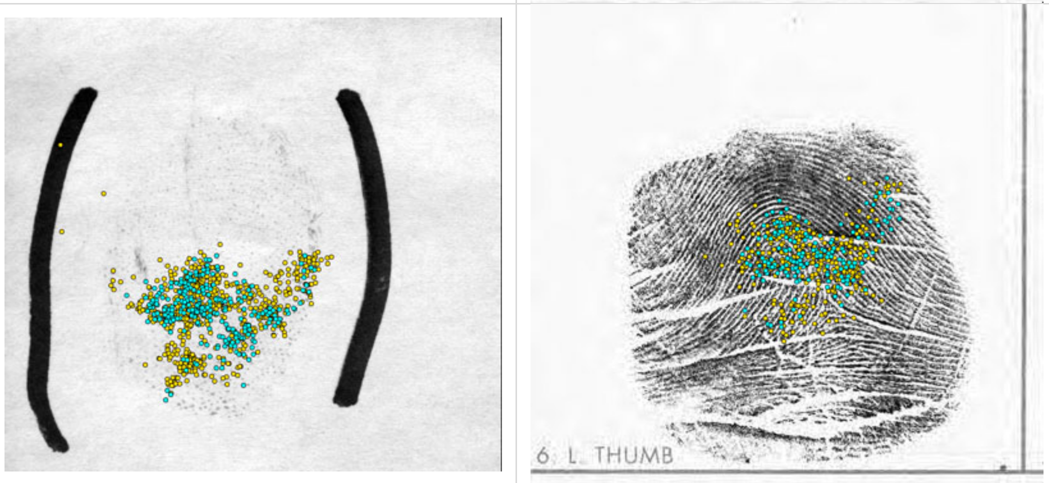	Bottom row: Inconclusive<br>Very Difficult<br>9.9 minute comparison time

Fig S13. Examples of fixations for three trials on a mated image pair (CW309). Overall conclusion counts: No Value 7, exclusion 4, inconclusive 13, identification 3, Total 27.

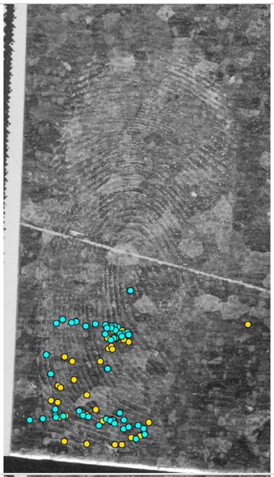

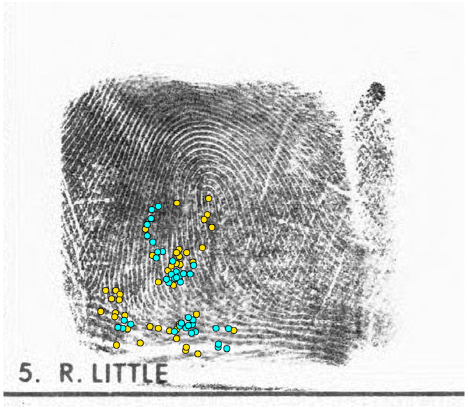

Top row: Identification (false positive)
Moderate
1.2 minute comparison time

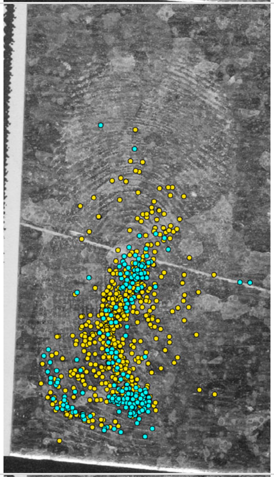

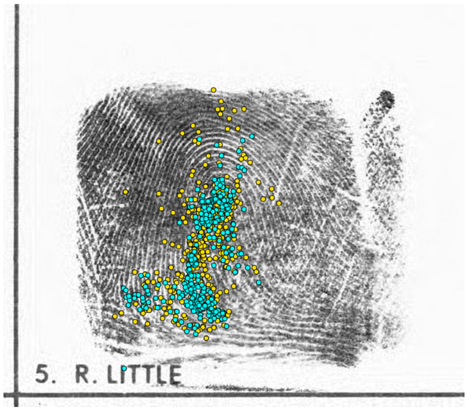

Middle row: Exclusion (borderline)
Difficult
9.7 minute comparison time

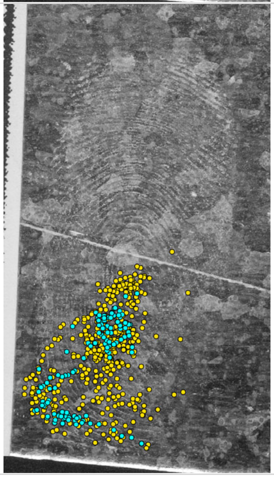

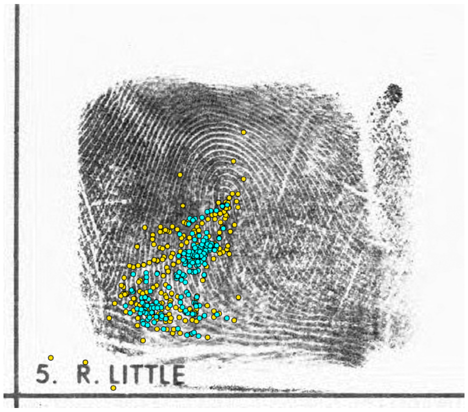

Bottom row: Exclusion
Moderate
6.5 minute comparison time

Fig S14. Examples of fixations for three trials on a nonmated image pair (CW447). Overall conclusion counts: Exclusion 19, identification 1, inconclusive 11, Total 31. The false positive trial is by an examiner other than the examiner discussed in Section 3.5 — none of the other false positive trials have releasable exemplar images (fingerprints are protected as Personally Identifiable Information and public release requires permission from the subject, which could not be obtained for those exemplars). Aqua points are detail/deciding fixations, and yellow points are scanning and miscellaneous fixations.

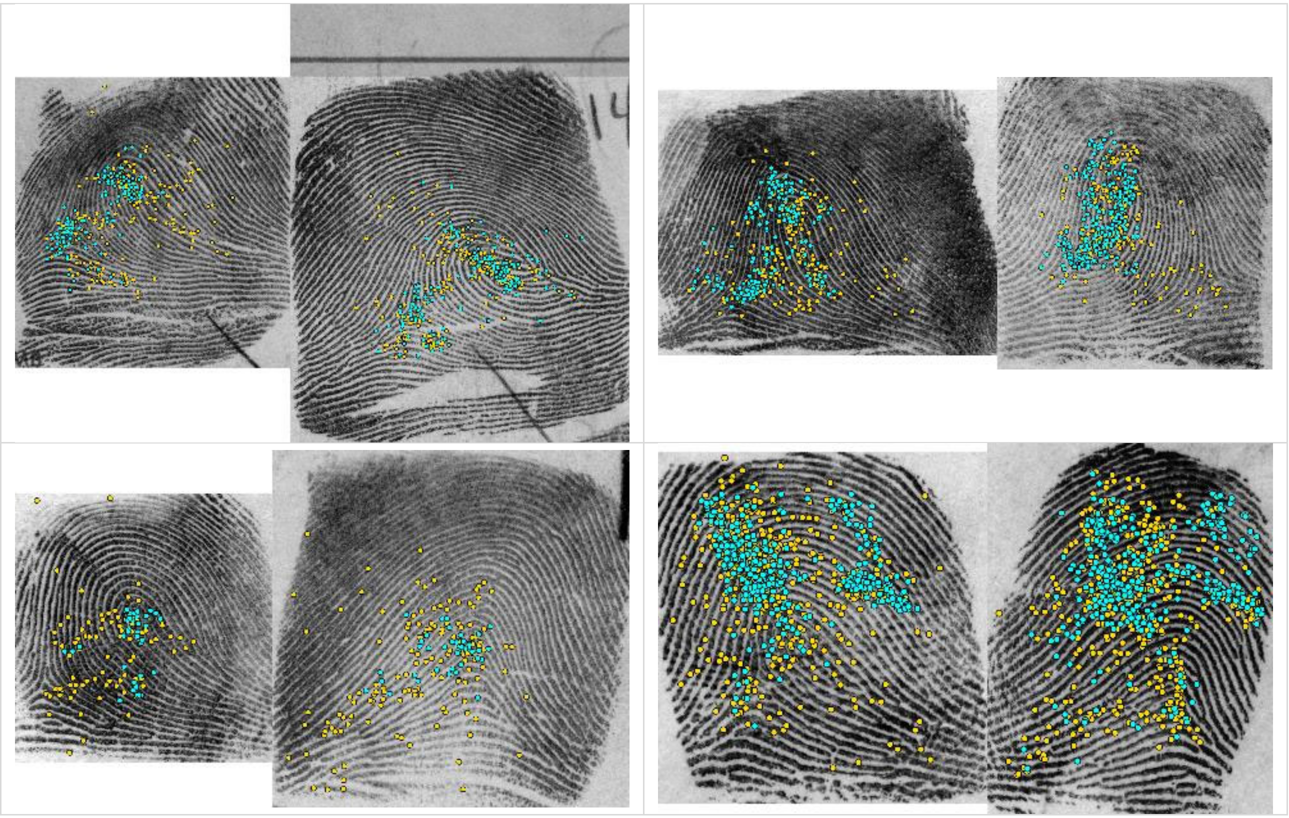

Fig S15. Raw fixations for four false-positive exemplar-exemplar trials by the participant discussed in Section 3.5; that examiner assessed the difficulty as moderate. No other participants made errors on exemplar-exemplar image pairs: all other conclusions on these image pairs were true negatives (exclusions) on which the difficulty was assessed as easy or very easy. Aqua points are detail/deciding fixations, and yellow points are scanning and miscellaneous fixations.
