# Supplementary material for: Characterizing missed identifications and errors in latent fingerprint comparisons using eye-tracking data
Source: PLoS One. 2021 May 24;16(5):e0251674. doi: 10.1371/journal.pone.0251674 (PMC8143401; doi:10.1371/journal.pone.0251674)
Supplement: S1 File — (PDF) [file pone.0251674.s005.pdf]

Characterizing missed identifications and errors in latent fingerprint comparisons using eye-tracking data

Supporting Information

Contents

APPENDIX SI-1 FINGERPRINT DATA DESCRIPTION .... ERROR! BOOKMARK NOT DEFINED.

APPENDIX SI-2 DISTRIBUTION OF OUTCOMES FOR ALL IMAGE PAIRS .... ERROR! BOOKMARK NOT DEFINED.

APPENDIX SI-3 METRIC DEVELOPMENT, TEST INSTRUCTIONS, AND ADDITIONAL ANALYSES .... ERROR! BOOKMARK NOT DEFINED.

APPENDIX SI-3.1 METRIC DEVELOPMENT .... **ERROR! BOOKMARK NOT DEFINED.**

Appendix SI-3.1a Fixation segmentation and drift correction .... ***Error! Bookmark not defined.***

Appendix SI-3.1b Fixation spread .... ***Error! Bookmark not defined.***

Appendix SI-3.1c Subphase determination .... ***Error! Bookmark not defined.***

Appendix SI-3.1d Spatial clustering .... ***Error! Bookmark not defined.***

Appendix SI-3.1e Temporal transition assignment and correspondence attempt estimation: The TECA model .... ***Error! Bookmark not defined.***

Appendix SI-3.1f KS test statistic .... ***Error! Bookmark not defined.***

APPENDIX SI-3.2 SUMMARY OF EYE-TRACKING STUDY TEST INSTRUCTIONS .... **ERROR! BOOKMARK NOT DEFINED.**

APPENDIX SI-3.3 ANALYSIS TIME .... **ERROR! BOOKMARK NOT DEFINED.**

APPENDIX SI-3.4 NUMBER OF FIXATIONS PRIOR TO SWITCHING IMAGES .... **ERROR! BOOKMARK NOT DEFINED.**

APPENDIX SI-3.5 IMAGE CLARITY .... **ERROR! BOOKMARK NOT DEFINED.**

APPENDIX SI-4 EXAMPLES OF FIXATION DATA .... ERROR! BOOKMARK NOT DEFINED.

REFERENCES .... ERROR! BOOKMARK NOT DEFINED.

Glossary

This section defines terms and acronyms as they are used in this paper.

**ACE-V**	The prevailing method for latent print examination: Analysis, Comparison, Evaluation, Verification.
**Analysis phase**	The first phase of the ACE-V method. In these studies, the examiner assessed the latent and made a value determination before seeing the exemplar print.
**Comparison phase (Comparison/Evaluation phase)**	The second and third phases of the ACE-V method. In this test, there was no procedural demarcation between the Comparison and Evaluation phases of the ACE-V method; hence, this refers to the single combined phase during which both images were presented side-by-side.
**Comparison determination**	The determination of identification, exclusion, or inconclusive reached in the Comparison/Evaluation phase of ACE-V.
**Conflict resolution**	The process conducted when there is a difference of determinations or conclusions between examiners, generally when the initial examiner and verifier disagree.
**Determination**	The result of an examiner's decision: the Analysis phase results in a Value determination, and the Comparison/Evaluation phase results in a Comparison determination.
**Exclusion**	The comparison determination that the latent and exemplar fingerprints did not come from the same finger.
**Exemplar**	A fingerprint from a known source, intentionally recorded.
**False negative (FN)**	An erroneous exclusion of a mated image pair by an examiner.
**False positive (FP)**	An erroneous identification of a nonmated image pair by an examiner.
**ID (identification)**	The comparison determination that the latent and exemplar fingerprints originated from the same source.
**Inconclusive**	The comparison determination that neither identification nor exclusion is possible.
**Insufficient**	When referring to examiner determinations (response data), "Insufficient" responses include both latent NV determinations (Analysis phase) and inconclusive determinations (Comparison/Evaluation phase).
**Latent (or latent print)**	An image of a friction ridge impression from an unknown source. In North America, "print" is used to refer generically to known or unknown impressions. Outside of North America, an impression from an unknown source (latent) is often described as a "mark" or "trace," and "print" is used to refer only to known impressions (exemplars).
**Mated**	A pair of images (latent and exemplar) known a priori to derive from impressions of the same source (finger). Compare with "ID," which is an examiner's determination that the prints are from the same source.
**Missed ID**	Failure by an examiner to identify a mated pair that was identified by a consensus of other examiners.
**Nonmated**	A pair of images (latent and exemplar) known a priori to derive from impressions of different sources (different fingers and/or different subjects).
**NV (No value)**	The impression is not of value for identification and contains no usable friction ridge information.
**Source**	An area of friction ridge skin from which an impression is left. Two impressions are said to be from the "same source" when they have in common a region of overlapping friction ridge skin.
**Sufficient**	An examiner's assessment that the quality and quantity of information in a print (or image pair) justifies a specific determination (especially used with respect to identification).
**True negative (TN)**	An exclusion of a nonmated image pair by an examiner.
**True positive (TP)**	An identification of a mated image pair by an examiner.
**Value determination**	An examiner's determination of the suitability of an impression for comparison: Of Value (sometimes divided into value for identification (VID) vs. value for exclusion only (VEO)), or No Value (NV).
**Verification**	The final phase of ACE-V: the independent application of the ACE process by a subsequent examiner to either support or refute the conclusions of the original examiner.
**VID**	Determination based on the analysis of a latent that the impression is of value and is appropriate for potential identification if an appropriate exemplar is available. See also VEO and NV.
**VEO**	Value determination based on the analysis of a latent that the impression is of value for exclusion only and contains some friction ridge information that may be appropriate for exclusion if an appropriate exemplar is available. See also NV and VID.
