# Supplementary material for: Characterizing missed identifications and errors in latent fingerprint comparisons using eye-tracking data
Source: PLoS One. 2021 May 24;16(5):e0251674. doi: 10.1371/journal.pone.0251674 (PMC8143401; doi:10.1371/journal.pone.0251674)
Supplement: S1 References — (PDF) [file pone.0251674.s007.pdf]

References

1. Ulery BT, Hicklin RA, Buscaglia J, Roberts MA. Accuracy and reliability of forensic latent fingerprint decisions. P Natl Acad Sci USA. 2011;108(19):7733-8. doi: Doi 10.1073/Pnas.1018707108. PubMed PMID: WOS:000290439500024.

2. Ulery BT, Hicklin RA, Roberts MA, Buscaglia J. Measuring what latent fingerprint examiners consider sufficient information for individualization determinations. Plos One. 2014;9(11):e110179. Epub 2014/11/06. doi: 10.1371/journal.pone.0110179. PubMed PMID: 25372036; PubMed Central PMCID: PMCPMC4221158.

3. Engbert R, Mergenthaler K. Microsaccades are triggered by low retinal image slip. P Natl Acad Sci USA. 2006;103(18):7192-7. doi: 10.1073/pnas.0509557103. PubMed PMID: WOS:000237399900073.

4. Port NL, Trimberger J, Hitzeman S, Redick B, Beckerman S. Micro and regular saccades across the lifespan during a visual search of "Where's Waldo" puzzles. Vision Res. 2016;118:144-57. doi: 10.1016/j.visres.2015.05.013. PubMed PMID: WOS:000368968600014.

5. Hicklin RA, Ulery BT, Busey TA, Roberts MA, Buscaglia J. Gaze behavior and cognitive states during fingerprint target group localization. Cogn Res. 2019;4. doi: ARTN 12 10.1186/s41235-019-0160-9. PubMed PMID: WOS:000463671000001.

6. Kundel HL, Nodine CF, Carmody D. Visual Scanning, Pattern-Recognition and Decision-Making in Pulmonary Nodule Detection. Invest Radiol. 1978;13(3):175-81. doi: Doi 10.1097/00004424-197805000-00001. PubMed PMID: WOS:A1978FE29600001.

7. Holder EH, Robinson LO, Laub JH. The fingerprint sourcebook: US Department. of Justice, Office of Justice Programs, National Institute of …; 2011.

8. Cheng YZ. Mean Shift, Mode Seeking, and Clustering. Ieee T Pattern Anal. 1995;17(8):790-9. doi: Doi 10.1109/34.400568. PubMed PMID: WOS:A1995RL03500005.

9. Ashbaugh DR. Quantitative-qualitative friction ridge analysis : an introduction to basic and advanced ridgeology. Boca Raton, Fla.: CRC Press; 1999. xiv, 234 p. p.

10. Goldstone RL, Rogosky BJ. Using relations within conceptual systems to translate across conceptual systems. Cognition. 2002;84(3):295-320. doi: Pii S0010-0277(02)00053-7 Doi 10.1016/S0010-0277(02)00053-7. PubMed PMID: WOS:000176306400003.

11. Ulery BT, Hicklin RA, Roberts MA, Buscaglia J. Interexaminer variation of minutia markup on latent fingerprints. Forensic Sci Int. 2016;264:89-99. doi: 10.1016/j.forsciint.2016.03.014. PubMed PMID: WOS:000378623200013.

12. Kalka ND, Hicklin RA. On relative distortion in fingerprint comparison. Forensic Sci Int. 2014;244:78-84. Epub 2014/09/13. doi: 10.1016/j.forsciint.2014.08.007. PubMed PMID: 25216456.

13. Virtanen P, Gommers R, Oliphant TE, Haberland M, Reddy T, Cournapeau D, et al. SciPy 1.0: fundamental algorithms for scientific computing in Python. Nature methods. 2020;17(3):261-72.

14. Hunter JD. Matplotlib: A 2D graphics environment. Computing in science & engineering. 2007;9(3):90-5.

15. Waskom M, Botvinnik O, O'Kane D, Hobson P, Lukauskas S, Gemperline DC, et al. mwaskom/seaborn: v0. 8.1 (September 2017). Zenodo, doi. 2017;10.

16. Seabold S, Perktold J, editors. Statsmodels: Econometric and statistical modeling with python. Proceedings of the 9th Python in Science Conference; 2010: Austin, TX.
